# *i*DocChip: A Configurable Hardware Accelerator for an End-to-End Historical Document Image Processing

**DOI:** 10.3390/jimaging7090175

**Published:** 2021-09-03

**Authors:** Menbere Kina Tekleyohannes, Vladimir Rybalkin, Muhammad Mohsin Ghaffar, Javier Alejandro Varela, Norbert Wehn, Andreas Dengel

**Affiliations:** 1 Microelectronic Systems Design Research Group, University of Kaiserslautern, 67663 Kaiserslautern, Germany; ghaffar@eit.uni-kl.de (M.M.G.); varela@eit.uni-kl.de (J.A.V.); wehn@eit.uni-kl.de (N.W.); 2 German Research Center for Artificial Intelligence (DFKI), 67663 Kaiserslautern, Germany; Andreas.Dengel@dfki.de

**Keywords:** OCR, hardware-software co-design, FPGA, Zynq, hardware architecture, image processing, historical documents

## Abstract

In recent years, there has been an increasing demand to digitize and electronically access historical records. Optical character recognition (OCR) is typically applied to scanned historical archives to transcribe them from document images into machine-readable texts. Many libraries offer special stationary equipment for scanning historical documents. However, to digitize these records without removing them from where they are archived, portable devices that combine scanning and OCR capabilities are required. An existing end-to-end OCR software called anyOCR achieves high recognition accuracy for historical documents. However, it is unsuitable for portable devices, as it exhibits high computational complexity resulting in long runtime and high power consumption. Therefore, we have designed and implemented a configurable hardware-software programmable SoC called *i*DocChip that makes use of anyOCR techniques to achieve high accuracy. As a low-power and energy-efficient system with real-time capabilities, the *i*DocChip delivers the required portability. In this paper, we present the hybrid CPU-FPGA architecture of *i*DocChip along with the optimized software implementations of the anyOCR. We demonstrate our results on multiple platforms with respect to runtime and power consumption. The *i*DocChip system outperforms the existing anyOCR by 44× while achieving 2201× higher energy efficiency and a 3.8% increase in recognition accuracy.

## 1. Introduction

In the modern age, information is often provided and circulated as digital data. Optical character recognition (OCR) is the most common method of transcribing typed, handwritten, or printed documents into digital format. It is used in many application areas to digitize document images like passports, invoices, and others. The conventional approach for OCR requires capturing document images using a standalone scanner and later recognizing these images using a software running on a computer. This approach is time consuming and not scalable. Alternatively, bulky scanning devices can be replaced by specialized hand-held devices, like smart pens [1,2,3,4,5,6] that are equipped with a scanner to scan texts line-by-line. Some of them are equipped with OCR functionality. However, their application is restricted to a single text line recognition at once. Although this approach provides portability, it is time-consuming for documents with multiple text lines and for large numbers of documents.

The progress in algorithms and electronics has allowed robust OCR that can rely on low-resolution images taken by embedded cameras, instead of high-quality scanned images. This potentially enables any device equipped with a camera to perform OCR. One approach to achieve text transcription on portable devices is using cloud OCR services, to name a few: Cloud Vision from Google [7], Computer Vision from Microsoft [8], ABBYY Cloud OCR SDK [9], and CloudOCR [10]. This approach enables OCR on any device with Internet connection. Cloud-based OCR saves energy by offloading the text recognition task to a server. However, this approach requires Internet connection and it is based on a service that requires costly subscription. Another approach addresses the shortcomings of the cloud-based method by using embedded intelligence. However, embedded central processing units (CPUs) and graphics processing units (GPUs) typical to portable devices fail to provide a low-power, low-latency, and energy-efficient solution for OCR. Application specific accelerators enable complex algorithms in low-power portable devices with restricted battery budget. Field-programmable gate array (FPGA) are emerging computing platforms that are used to accelerate applications on portable devices. Recently, FPGAs started appearing in mainstream smartphones, like iPhone from Apple [11].

To this end, we propose an *i*DocChip that is a low-power and energy-efficient hardware-software accelerator suitable for a low-latency OCR. The *i*DocChip allows end-to-end OCR capable of producing a machine readable text from a camera image. In this paper, we target OCR of historical documents as they are one of the most challenging types of documents to transcribe, due to their complex layout and the various types of degradation they incur.

National archives, libraries, and museums worldwide hold millions of historical documents containing rich, diverse, and valuable information. These documents are usually very fragile due to physical deterioration. Digitizing these archives is essential to capture and preserve the history and information they contain. There has been an increasing demand to electronically access historical records. Converting archives into machine-readable texts has many benefits; mainly, it enables document indexing for easy identification, storage, and retrieval of information. Moreover, it leads to the development of further applications such as text mining, keyword spotting, text-to-speech conversion, and others. Over the past few decades, therefore, the research area to transcribe historical document contents into machine-readable texts has been amplified. Similarly, a searchable historical record is achieved by applying OCR to scanned archive pages.

Nowadays, libraries offer highly specialized equipment to scan historical documents. These machines are able to scan a wide range of document types with varying thicknesses and dimensions. However, they are usually large and stationary, requiring physical manipulations of the archives. To avoid further damage to the already fragile historical documents, using a portable device that combines scanning and OCR capabilities has become a promising approach. Such a device enables historical records to be transcribed without the need to remove them from where they are archived.

In support of the ongoing digitization efforts, we have focused on developing an OCR for a handheld device that is suitable for transcribing historical as well as contemporary modern documents. One of the applications that we envision for our system is integration with smart goggles [12,13,14,15] that will enable instant OCR for keyword spotting and similar tasks. Further, our system can be used as an intellectual property (IP) and integrated to embedded devices to perform OCR at low power consumption and high energy efficiency. The processing latency and power consumption requirements are formally set to design an efficient system that meets the thermal design power (TDP) constraints of a portable device. The resulting handheld OCR device is expected to transcribe a single document image within 500 ms under a 2 W power budget. These constraints can be further translated into 2 frames per second (FPS) throughput and 1 FPS/W energy efficiency. Additionally, the device is required to transcribe different historical and modern documents with high accuracy.

In recent years, various commercial and open-source OCR systems, such as ABBYY [16], OmniPage [17], OCRopus [18], Tesseract [19], and others, have been developed. These systems are typically optimized for transcribing modern documents, and they struggle to transcribe deteriorated historical archives with sufficient accuracy. Low-quality OCR text output reduces search efficiency, information retrieval, and other applications. Hence, to fully realize the benefits of digitization, an OCR system should offer high recognition accuracy, despite the severe quality degradation existing in historical documents. To this end, Bukhari et al. [20] introduced an open-source end-to-end OCR software called anyOCR. This digitization solution is adopted by Nararagonien-digital [21]. The digitization project [22] has been conducted by the German Research Centre for Artificial Intelligence [23] in collaboration with the University of Würzburg [24].

Unlike many commercial and open-source OCR engines, the anyOCR system transcribes modern and historical documents with high accuracy. It is designed using state-of-the-art image processing techniques, with a particular emphasis on digitizing historical document images that suffer from severe quality degradation caused due to bleed-through pages, complex irregular layouts, skewed/overlapping texts, non-uniform shading, etc. Such degradations are typical to historical documents, which makes them distinct from contemporary documents. In this paper, we have used a highly degraded historical Latin document images dataset, which is part of the 15th century novel called “Narrenshchiff” [25]. This dataset is used as a template to design our system as it represents the type of historical documents we target. For this test dataset, the commercially available OCR engine ABBYY and the open-source Tesseract system achieve only 66.47% and 56.83% accuracy, respectively. In comparison, anyOCR achieves a 76.3%. Therefore, we selected the algorithm of the anyOCR system for our portable OCR device. However, due to a large number of sophisticated image processing techniques, anyOCR exhibits a high computational complexity that results in a long runtime and high power consumption. Therefore, to adapt anyOCR for a portable device with a constrained energy budget, we target energy-efficient and high-throughput acceleration of the anyOCR algorithm.

The first crucial step in the design of a portable OCR device is selecting a computing platform that best fits the anyOCR system. General-purpose processing platforms that rely on traditional arithmetic, like CPUs and GPUs, support only a limited number of data types. For systems like anyOCR that require many arbitrary-bit operations, computing using standard data types significantly affects their energy efficiency. As a result, anyOCR cannot be handled efficiently on general-purpose computing platforms. On the contrary, hardware platforms like FPGAs and application-specific integrated circuits (ASICs) support a full range of data types, including custom precision. Hence, these platforms allow the development of efficient architectures that can benefit from arbitrary precision operations. Additionally, these specialized processors offer customized hardware acceleration, custom memory hierarchy, and other features, which enable the design of systems that can achieve low power and meet real-time processing requirements. However, ASICs are inherently inflexible and are reasonable only for high volume production due to large-scale manufacturing costs and high design efforts. Moreover, to support various document recognition applications with different configurations, the portable OCR engine has to adapt to changes even after the production of the device, which is not possible with ASICs. To this end, FPGAs is the most suitable design platform as it fulfills all the requirements. In recent years, FPGA vendors have introduced a System-on-Chip (SoC) that offers software, hardware, and I/O programmability in a single chip. The Xilinx^®^ Zynq^®^-7000 All Programmable SoCs leverage the 28 nm FPGA fabric and the ARM^®^ Cortex^™^-A9 dual-core processors to increase design flexibility and enable the development of highly versatile systems. Hence, we target the Zynq-7000 SoC, specifically the Zynq-7045 device, to design the anyOCR-based portable document image processor, *i*DocChip. Furthermore, the Zynq platform enables hardware upgrade in a similar manner to the software, which allows for the updating of the complete system with newer algorithms.

**Figure 1:**
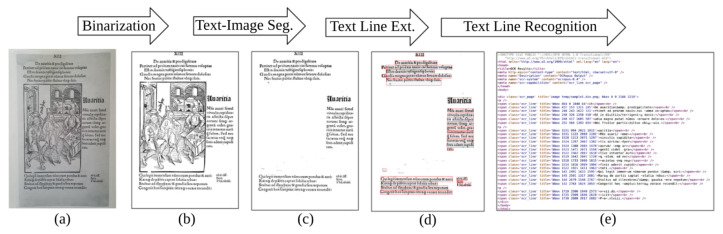
The anyOCR system processing pipeline: (**a**) input image, (**b**) after Binarization, (**c**) after Text and Image Segmentation, (**d**) after Text Line Extraction, and (**e**) after Text Line Recognition.

As shown in Figure 1, anyOCR has four pipeline steps. The first three are preprocessing/layout analysis steps, namely, Binarization, Text and Image Segmentation, and Text Line Extraction, which involve various computer vision and image processing algorithms. The last pipeline step, Text Line Recognition, is a character recognition step based on a Bidirectional LSTM (Bi-LSTM) recurrent neural network. While the character recognition step of anyOCR is universal and suitable for both contemporary and historical documents, the binarization and layout analysis steps are designed with a special emphasis on historical documents, as the historical documents are typically more physically deteriorated than contemporary documents and have complex layout structure.

In previous publications [26,27,28,29,30], we have presented hardware architectures for each of these four pipeline steps. However, these architectures are separately developed and require modifications to put all pipelines together. Moreover, the assembly of several filters, computer vision algorithms, and the recognition network have resulted in new challenges, such as data storage management. In the previously presented implementations, each pipeline step reads data from off-chip memory. This work focuses on coupling the different pipelines together in a single design, which requires keeping a large amount of data on-chip and reducing external memory transfers to achieve real-time processing and low-power consumption. As a result, a new hardware-software partitioning scheme is required.

Our test dataset [25] is composed of two types of images: (1) high-resolution scanned images with dimensions of 2166×3219 and 400 ppi, (2) lower resolution images with dimensions of 4248×5664 with 72 ppi taken by a smartphone camera (Samsung Galaxy A9). We took the camera images by holding the phone parallel to the printed document, without any mechanical support. Holding the phone by hand has resulted in images with varying skew angles and perspective distortions for different document pages. Moreover, the smartphone camera has exhibited noises, such as variations in the brightness of the images. Similar to the images acquired from the scanner, we do not apply any extra preprocessing for images taken by the smartphone camera. The binarization part of the iDocChip system is able to mitigate the camera noises and other unwanted artifacts. We expect images taken by any regular user without any photography experience. This way, the test document images are in accordance with a real-world scenario, where the images taken by an embedded camera are fed to our iDocChip to produce a machine-readable text.

Input images of the *i*DocChip system may have a high resolution and big size. To avoid the high bandwidth overhead and meet the throughput constraints, the data transfers between computational units and off-chip memory must be limited to the minimum possible value. This implies that intermediate results of operations in *i*DocChip must be buffered on on-chip memory units. However, due to the limited number of available on-chip memory units, storing all intermediate results using hardware resources is not feasible, even for the largest available FPGA fabric. To relieve the high bandwidth overhead and large memory requirements, we have analyzed different hardware-software partitioning schemes. This paper presents a hybrid hardware-software architecture for the complete iDocChip system that uses an optimized and efficient hardware-software partitioning scheme. The capability of the programmable fabric and the embedded CPU is exploited to design a low-power, energy-efficient, real-time, adaptable end-to-end OCR system. By setting *i*DocChip with different configuration files, different types of documents can be transcribed. Hence, this system enables the digitization of a wide range of documents. The novel contributions of this paper are as follows:

- Algorithmic optimizations for the anyOCR system are presented that improve the accuracy of the historical document digitization by 3.8%.
- A new hardware-software partitioning scheme is presented for the optimized anyOCR algorithm.
- A heterogeneous hardware-software architecture is designed and implemented based on the new partitioning scheme.
- A custom hardware accelerator based on the new architecture is realized using Zynq-7045 FPGA.
- The novel accelerator is compared to optimized anyOCR implemented on multiple computing platforms, including low-power CPUs.
- It is demonstrated that the *i*DocChip system outperforms the original anyOCR software running on i7-4790T by more than 44× and 2201× in terms of runtime and energy efficiency, respectively.

The rest of the paper is organized as follows. Section 2 reviews related works in the literature. Then in Section 3 and Section 4, the anyOCR algorithm and our previous works with respect to *i*DocChip are explained, respectively. In Section 5, algorithmic optimizations and the updated hardware-software partitioning for the end-to-end *i*DocChip are described in detail. Then the hybrid architecture of *i*DocChip is presented in Section 6, followed by evaluation and results in Section 7. Finally, Section 8 gives an outlook and concludes the paper.

## 2. Related Works

To develop a portable OCR device, three major design decisions are considered: platform, algorithm, and implementation. Hence, this section reviews existing literature with respect to these main topics. Regarding the computing platform, the design space includes embedded CPUs, embedded GPUs, FPGAs, and ASICs. To choose the most suitable computing platform for designing the handheld OCR device, we surveyed existing works in the literature that compare portable design platforms for image processing algorithms, as presented in Section 2.1. Then Section 2.2 reviews algorithms of end-to-end OCR systems that are available in the literature. Finally, published works concerning hardware implementation of OCR systems are explored in Section 2.3.

### 2.1. Cross-Platform Comparisons

Targeting image processing algorithms, many publications have presented comprehensive comparisons of different processing platforms [31,32,33,34,35]. Brugger et al. [31] have presented a cross-platform analysis of morphological operations implemented on low power platforms: a Low-Power Intel^®^ Core^™^ i7-4790T CPU, an NVIDIA^®^ Tegra^®^ K1 GPU SoC, and a Xilinx^®^ Zynq^®^ 7020 FPGA SoC. The authors observed that the filtering algorithms implemented on the GPU are 5× slower than a similar implementation on the CPU, while the FPGA implementation is 8–10× more energy efficient compared to the CPU and GPU implementations. In another publication [32], Qasaimeh et al. benchmarked performance and energy efficiency for different vision algorithms implemented on three hardware accelerators that are commonly used for embedded vision applications: ARM^®^ Cortex^™^-A57 CPU, NVIDIA^®^ Jetson^™^ TX2 GPU, and Xilinx^®^ Zynq^®^ UltraScale+^®^ MPSoC ZCU102 FPGA. Their results show that while simple and easy-to-parallelize kernels perform well on GPUs with 1.1–3.2× energy/frame reduction compared to CPU and FPGA, for complete vision pipelines the FPGA outperforms the others with energy/frame reduction of 1.2–22.3×. Moreover, the authors also observed that the FPGA performs increasingly better as the complexity of the vision pipeline grows. In [33], Page and Mohsenin have presented a pulse wave spectral Doppler ultrasound imaging system implemented on a Xilinx^®^ Virtex^®^-5 FPGA and in a 65 nm CMOS ASIC. The authors concluded that the FPGA design has comparable energy efficiency and performance compared to the ASIC implementation while providing reconfigurability and lower costs.

According to our survey, FPGAs are the most suitable computing platforms to develop a cost-effective image processing system. The Xilinx^®^ Zynq^®^-7000 All Programmable SoC extends the FPGA fabric with a CPU; therefore, software programmability is possible due to the integrated dual-core ARM^®^ Cortex^™^-A9 processors, while the 28 nm Artix^®^-7/Kintex^®^-7 based programmable logic (PL) hardware provides reconfigurability. The processor cores, referred to as the processing system (PS), increase the flexibility of a design. Hence, together with the PL, the PS provides a highly customizable SoC. As a result, these hybrid SoCs enable highly differentiated designs for a wide range of embedded applications, including medical endoscopes, professional cameras, machine vision, and many others [36,37,38,39]. In particular, the Xilinx Zynq-7045 SoC, which contains the Kintex-7 based PL alongside the ARM cores, is used for diverse portable industrial applications [40,41,42]. Hence, we have selected this Xilinx Zynq SoC as a target platform to design the portable OCR device.

### 2.2. End-to-End OCR Systems

Typically, OCR is carried out in four phases: image enhancement, page segmentation, feature extraction, and character recognition (classification). A post-processing step, e.g., a language model, may also be included to increase the character recognition accuracy of the system. First, preprocessing is performed on the input image to make it ready and suitable for character classification. The preprocessing phase enhances the input image, removes any existing noises, segments the page into appropriate groups, detects, and extracts features. Then the classifier module labels characters through supervised learning. Finally, post-processing is performed to increase the overall system accuracy by correcting any existing errors after the OCR process.

In the literature, there exist several works regarding modern contemporary and historical document processing systems. In [43], Afroge et al. preprocessed input images using different image processing algorithms. Then they use a feed-forward neural network for classifying and recognizing the characters. However, their method cannot distinguish between text and non-text in the document image. In addition, it is unable to extract connected characters, making it unsuitable for digitizing historical documents. Wei et al. [44] proposed an OCR model composed of four major blocks: input acquisition and preprocessing, training, testing, and validation. For character recognition, the authors have used a pre-trained deep neural network Inception V3 model with two fully connected layers that give a 90% accuracy for broken and faded English characters. For classification (character recognition), researchers have also worked on different machine learning approaches, which include support-vector machine (SVM) [45], random forests [46], k-nearest neighbor [47], decision tree [48], neural networks [49,50,51] etc. These machine learning methods are usually combined with image processing techniques to increase the accuracy of the optical character recognition system. Nowadays, the pre- and post-processing steps are also being processed using neural networks [52,53,54]. Although these methodologies are very promising, due to the large number of parameters, they are very challenging to translate into hardware-aware implementations without loss of accuracy, especially for portable devices with real-time requirements.

The anyOCR system [20] is based on the open-source OCRopus toolbox [18], which is the first OCR engine to implement a line-based character recognition using bidirectional long short-term memory (LSTM) networks [55]. The anyOCR chain implemented in Python is comprised of various algorithms for document analysis and recognition, including binarization, page segmentation, text line extraction, and character recognition modules. The preprocessing achieves very high results due to the robust deskewing and adaptive thresholding techniques used for binarization. The text and image-parts of the documents are separated using page segmentation. The robust text line extraction reliably identifies text lines within the document and outputs these lines in reading order. Moreover, the Bi-LSTM network has a very high character recognition accuracy.

### 2.3. End-to-End OCR Hardware Architectures

Many publications present hardware acceleration for the different steps of the OCR system. For example, Singh et al. [56] and Chen et al. [57] presented GPU-based parallel implementations that speed up Souvola’s method of binarization. In [58], Singh et al. presented a parallel implementation for Otsu’s method of binarization, which outperforms the serial implementation by 1.6×. Soua et al. [59] proposed parallel implementation of the hybrid binarization based on Kmeans method on the NVIDIA GTX 660 GPU. Westphal et al. [60] implemented Howe’s binarization algorithm on a heterogeneous CPU-GPU system and achieved an average of 3.5× faster performance compared to a CPU-only execution. In [61], Sultana and Meenakshi developed an image binarization algorithm that used a simple two-weight neural network-based clustering and implemented this algorithm on an FPGA. Recently, Rybalkin and Wehn [62] presented a hardware architecture for multidimensional long short-term memory (MD-LSTM) neural network and its FPGA accelerator for image binarization.

In the literature, text line extraction is usually coupled with text and image segmentation. In [63], Kumar et al. used a method based on a discrete wavelet transform to detect and extract texts from document images. The authors designed the architecture and implemented the system on a Virtex-5 FPGA. For a dataset of 33 images, the authors achieved 96 s for the text and image segmentation process. However, they have not openly communicated the energy efficiency or power consumption of the system. Bai et al. [64] proposed a novel architecture of a convolutional neural network called MSP-Net for text/non-text image classification. The system takes an input image and outputs block-level classification results in an end-to-end manner. An NVIDIA GTX TitanX GPU was used for training purposes. In [65], Vignesh et al. applied a morphological closing operation and connected component analysis to detect and extract texts from an image. The authors used a Virtex7 FPGA device to synthesize and evaluate their architecture.

In recent years, different hardware architectures have been presented for the character recognition task. In [66], Sanni et al. presented a hardware implementation of a deep belief network architecture for character recognition using stochastic computation. The authors evaluated their architecture on a Kintex-7 FPGA device for the MNIST database of handwritten digits [67]. In the literature, some works combine feature extraction and character recognition. For example, Zho et al. [68] presented an OCR system that consists of character segmentation and recognition modules. The authors used a five-layer Convolutional Neural Network (CNN)-based recognizer. The system is implemented and tested on a CME M7 FPGA device. In [69], an FPGA-based hardware accelerator is presented for scene text recognition. The system involves feature extraction based on histogram of oriented gradients and character recognition based on an Extreme Learning Machine (ELM) feedforward neural network. The authors have used Altera Cyclone IV FPGA to prototype and evaluate the system.

To the best of our knowledge, there is no previously published work presenting a hardware architecture for an end-to-end OCR system in the domain of historical documents. Moreover, none of the previous publications targeted embedded platforms or focused on designing low-power real-time systems. Although in our previous publications [26,27,28,29,30], we have presented hardware architectures for each of the four OCR pipelines (see Section 4), these implementations are optimized for their corresponding step, and they require changes to integrate them into a single system.

## 3. The anyOCR Algorithm

**Figure 2:**
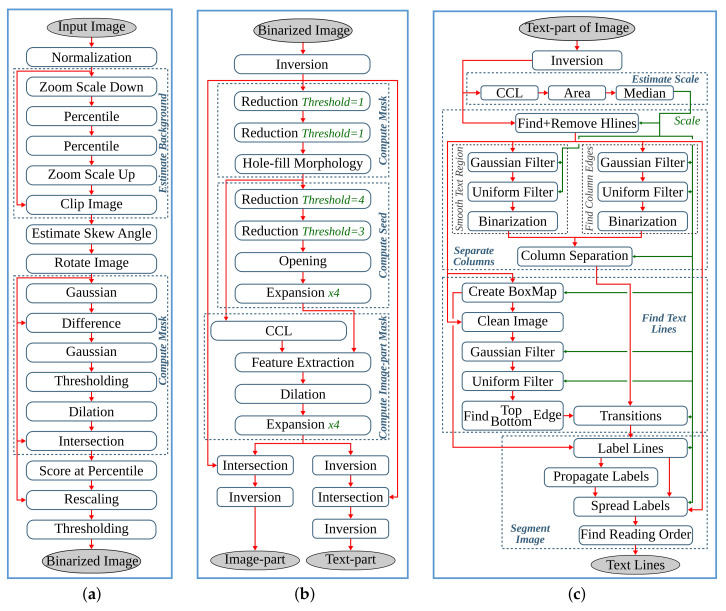
Algorithms of the three anyOCR preprocessing steps: (**a**) Binarization, (**b**) Text and Image Segmentation, and (**c**) Text-line Extraction.

The anyOCR system takes a scanned document page as an input. This image is prepared for the character recognition step by first passing through the preprocessing steps. As shown in Figure 2, the three preprocessing steps of the anyOCR algorithm consist of various image processing operations, including many filters that make use of a sliding window. The window size, also known as the kernel size or structuring element (SE), determines the scale of the image filtered at a time.

As shown in Figure 2a, the binarization step involves normalization, noise detection and removal, thinning, skew detection and correction, thresholding, and other processes. In the text-image segmentation step, Figure 2b, page segmentation is performed to identify patterns of non-text regions from the document and segment these areas using feature extraction. The third pipeline step of the anyOCR algorithm is based on Gaussian smoothing, which provides high accuracy for text line detection. The complete processing chain of the text line extraction algorithm is depicted in Figure 2c.

### 3.1. Binarization

The state-of-the-art results in historical document image binarization are achieved by methods based on U-Net fully convolutional network. The U-Net architecture was first proposed as a network for biomedical image segmentation [70]. Since then it became very popular in various other semantic segmentation tasks, including document image binarization [71,72,73,74]. The differences between the proposed methods come from the different architectural enhancements, the number of networks, a patch size, and operation on a local or/and global scale. The top result in Document Image Binarization Competition (DIBCO) [71] is achieved by a method proposed by Huang et al. in [74]. They proposed performing document image binarization using three U-Net networks. Combining outputs from all three networks, they could achieve superior results. However, the use of three original U-Net networks resulted in a very large model compared to previous approaches. Karpinski et al. in [73] proposed a U-Net architecture that achieves comparable accuracy to the model proposed by Huang et al., while having 122 times fewer parameters. They enhanced their architecture with residual connections and Squeeze-and-Excitation module [75], which have been shown to improve the performance. The document image binarization deployed in the anyOCR is based on percentile-based binarization (PBB) approach. This method is based on conventional image processing techniques as explained below. In Table 1, we compare the character-level accuracy after binarizing the document images using the three methods.

The size of the model proposed by Huang et al. [74] is prohibitively large for embedded implementation. Considering an 8-bit quantization of the model proposed by Karpinski et al. [73], only the model would require 31% of on-chip memory resources on Zynq-7045, which is 3.4 times higher than the corresponding utilization of the PBB method, see [26]. Considering the size of the models and the achieved OCR accuracy, PBB approach is the most suitable for the hardware implementation. The OCR accuracy is based on character error rate (CER) computed as the Levenshtein distance [76] between a decoded sequence and a ground truth after the character recognition step, see Section 3.4.

To binarize the input grayscale image $I_{input}$, first, it is normalized, where each pixel is processed using Equation (1). $P_{max}$ and $P_{min}$ are the maximum and minimum pixel values of $I_{input}$.

(1) $$P_{norm}=\left(P_{input}-P_{min}\right)/\left(P_{max}-P_{min}\right)$$

The *estimate background* block (see Figure 2a) involves five operations. First, the normalized image size is scaled down using cubic spline interpolation to reduce the number of pixels within the image. Then the shadow background layer of the image is estimated by applying consecutive *percentile filters*. After processing these filters, the image $I_{perc}$ is obtained. This image is then scaled back up to the original image size using third-degree *spline interpolation*. To further approximate the background layer, $I_{perc}$ is clipped as shown in Equation (2). These operations result in a clipped image $I_{clip}$.

(2) $$I_{clip}=\left\{\begin{array}{cc} 0, & (I_{norm}-I_{perc})<0\\ 1, & (I_{norm}-I_{perc})>1\\ (I_{norm}-I_{perc}), & otherwise \end{array}\right.$$

Computing the skew angle involves several operations, including maximum $P_{max}$ and minimum $P_{min}$ pixel value calculations, subtraction, eight nearest-neighbor based image rotation interpolations, arithmetic means, and variance computations. Finally, the angle with the maximum variance is selected as the skew angle. Then the image is rotated with the resulting skew angle by applying *spline interpolation* and normalized by subtracting each pixel of the image from the maximum pixel value. This processing step results in a rotated clip image $I_{rclip}$. The mask image is used to eliminate pixels that are not required for the final thresholding. As shown in Figure 2a, the *mask computation* block involves two *Gaussian filters*, *subtraction* of the first Gaussian output from the rotated clip image, *thresholding*, a *morphological dilation* with an 8-connective SE, and an *intersection* operation between the rotated clip image $I_{rclip}$ and the output of the dilated image. To generate the final binarized image, the resulting image of the *compute mask* block is flattened into a one-dimensional array, and all of its elements (pixels) are sorted. Next, the low ls and high hs scores are calculated at the 5th and 90th percentile positions, respectively. These values are used to rescale the rotated clip image $I_{rclip}$ as shown in Equation (3). Finally, the rescaled image is binarized using a thresholding operation to obtain the final binary image $I_{binary}$.

(3) $$I_{rescale}=\left\{\begin{array}{cc} 0, & \left(I_{rclip}-ls\right)/\left(hs-ls\right)<0\\ 1, & \left(I_{rclip}-ls\right)/\left(hs-ls\right)>1\\ \left(I_{rclip}-ls\right)/\left(hs-ls\right), & otherwise \end{array}\right.$$

### 3.2. Text and Image Segmentation

The input image of the text and image segmentation step is the binarized image $I_{binary}$. The foreground and background pixels of this binary image are represented by ′0′ s and ′1′ s, respectively. In order to make any valuable computations on the foreground image, first, the image is inverted, where its foreground image pixels are set to ″1″ s and background pixels are set to ″0″ s.

The inverted image $I_{inv}$ is then processed by two *reduction* operations with thresholds T=1. These operations subsample the incoming image while preserving the density of low- and high-frequency components within the image. After reading a 2×2 block of four pixels, a *reduction* operation replaces these pixels by a single pixel depending on the chosen threshold *T*, according to the formula given in Equation (4). The value of *T* can be between 1 and 4.

(4) $$P_{reduction}^{T}=\left\{\begin{array}{cc} 1, & \sum_{i=1}^{4}P_{i}\geq T\\ 0, & otherwise \end{array}\right.$$

The subsampled image is processed with a *hole-fill morphology* to fill hollow contours. This operation is based on an 8-connective *morphological reconstruction by erosion*. Then to compute the seed image (see Figure 2b), two more *reduction* operations of threshold values T=4 and T=3, a 4-connective *morphological opening*, and two *expansion* operations are applied. An *expansion* operation scales up an image by populating one pixel into a 2×2 block of four pixels. To compute the mask for the image-part of the document, first, connected component labeling (CCL) is applied on previously obtained $I_{mask}$, refer to Figure 2b. This operation finds and labels all distinct components within $I_{mask}$. Then, the labeled mask image $I_{mask\_labeled}$ is intersected with the seed image $I_{seed}$, and the labels of the connected components in $I_{mask\_labeled}$ that fully or partially overlap to the seed image are selected and collected as uniqueLables. In the last step of this *feature extraction* operation, the mask image is indexed by the uniqueLabels and unified with the seed image, as shown below.

(5) $$I_{feature\_ext}=\left\{\begin{array}{cc} 1, & I_{mask\_labeled}\in uniqueLables\\ I_{seed}, & otherwise \end{array}\right.$$

In the next processing step, an 8-connective *morphological dilation* is applied to recover any valuable pixels that were previously filtered out as noise. Then the original image size is retrieved by applying two more *expansion* operations. The resulting image, $I_{ip\_mask}$, contains the mask image for the non-text (image) part of the inverted binary image $I_{inv}$. The mask image for the text-part $I_{tp\_mask}$ is obtained by inverting $I_{ip\_mask}$. Then the final text- and image-parts of the input binary image, $I_{tp}$ and $I_{ip}$, respectively, are gathered by intersecting the inverted binary image $I_{inv}$ with their respective masks, $I_{tp\_mask}$ and $I_{ip\_mask}$, and inverting the results, as shown in Figure 2b.

### 3.3. Text Line Extraction

This step processes the text-part of the document image ($I_{tp}$) that was segmented in the previous processing pipeline. Initially, the image is inverted to give $I_{tp\_inv}$. Then the *estimate scale* block (see Figure 2c) approximates the text size of the document by first applying a CCL on the inverted image, extracting the bounding boxes of the connected components (CCs), and computing their area. Then *ScaleMap* (area map) is created by replicating the labeled $I_{tp\_inv}$ image and updating it by replacing the label of each pixel by the square root of its CCs’ corresponding area. The resulting area map is flattened into a one-dimensional array, and thresholding is applied to remove *Scales* that are too small or too large. Finally, the approximate text size (Scale) is selected by calculating the median of the one-dimensional *ScaleMap*. This Scale is used to determine the kernel sizes of the subsequent Gaussian, uniform, maximum filters, and different threshold values.

The second block of the text line extraction pipeline, *Separate Columns*, takes in the inverted image of the text-part of the document and outputs an image with its existing columns marked. This block is further divided into four sub-blocks, refer Figure 2c. The first sub-block finds horizontal lines that usually interface in images due to reflections and shadows. To prevent these lines from interfering with the text line extraction, they are detected and removed by applying CCL on the inverted $I_{tp\_inv}$ image and extracting, calculating, and removing objects with a width larger than a given threshold. The resulting $I_{hl}$ image is then broadcasted into the next two sub-blocks. These *smooth text region* and *find column edges* sub-blocks consist of Gaussian and uniform filters followed by binarization operations. The kernel sizes of the filters are dependent on the previously obtained text *Scale*. Unlike the *smooth text region* sub-block, to *find column edges*, an x-derivative of Gaussian is used. The two sub-blocks result in $I_{st}$ and $I_{ce}$ images after smoothing the text region and finding the column edges of the document, respectively. The fourth sub-block, *column separation*, involves seven operations: two maximum filters, comparison between images, a CCL that extracts the height of CCs, thresholding, and indexing. The threshold value and kernel sizes of the maximum filters are dependent on the value of *Scale*.

The *find the text lines* block involves different operations, see Figure 2c. First, a boxmap is created by applying a CCL on the $I_{hl}$ image to find the connected components. Then the area of each object is computed, and based on a given threshold, objects that are too small or too large are discarded, as they are not considered as characters. These threshold values are dependent on the Scale. The boxmap image $I_{bm}$ is then intersected with $I_{hl}$ in order to clean the image, where only the desired components that contain texts are kept. Gradient filtering is used to find baselines of the text edges by applying a y-derivative of Gaussian filter followed by a uniform filter and results in $I_{grad}$ image. This image is further processed to find the top and bottom edges of the text lines using the following equation:

(6) $$I_{top}=\left\{\begin{array}{cc} I_{grad}/max\left(I_{grad}\right), & I_{grad}>0\\ 0, & otherwise \end{array}\right.\;I_{bottom}=\left\{\begin{array}{cc} -I_{grad}/max\left(-I_{grad}\right), & I_{grad}<0\\ 0, & otherwise \end{array}\right.$$

Then the *transitions* operation finds the text lines of the document by applying several operations on the top and bottom edge images. It involves six maximum filters, two thresholding operations, an image inversion operation, search and fill area computations, and inter-image operations of three intersections and two multiplications. The *transitions* operation results the text-part of the document image with its text lines marked, $I_{lines}$.

The final processing block, *segment image*, involves four operations, refer to Figure 2c. First, the text lines of $I_{lines}$ and the boxmap image $I_{bm}$ are labeled using CCL, resulting in $I_{labeled\_lines}$ and $I_{labeled\_bm}$, respectively. Then in the *label propagation* operation, the corresponding labels of $I_{labeled\_bm}$ and $I_{labeled\_lines}$ are identified. Through indexing, the labels from $I_{labeled\_lines}$ are propagated to $I_{labeled\_bm}$ in such a way that if a character intersects exactly one text line, it is kept, and the label of this character is set similar to the label of its text line. However, characters that intersect more than one text line are considered as strokes caused by quality degradation, and therefore, they are removed. The output image $I_{propagate}$ contains labeled text lines. The *spread labels* operation reassigns labels that were wrongly removed in the label propagation step by applying an Euclidean Distance Transform (EDT), three thresholding, two image-multiplication, an indexing, and two flattening operations. Finally, the text lines are segmented by applying CCL and extracting the connected component objects. Then the text lines are sorted in a reading order using topological sort algorithm.

### 3.4. Text Line Recognition

**Figure 3:**
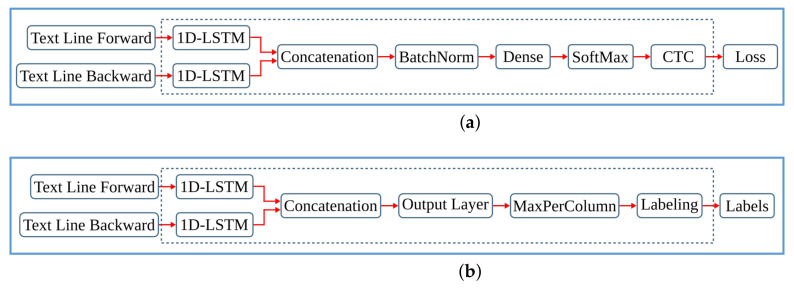
(**a**) Network topology used during training. Only forward path is shown. (**b**) Network topology used during inference.

The last step of the anyOCR chain, text line recognition, is based on Bi-LSTM neural network with connectionist temporal classification (CTC) that allows transcribing the text lines without partitioning them down to separate characters. As shown in Figure 3, the topologies used for training and inference have differences. Figure 3a depicts a topology used for training, while Figure 3b presents a modified topology used during inference.

First, we explain the topology used for training. The input text lines are scaled to have a fixed height of $N^{I}=48$ pixels and arbitrary width of *C* pixels. The height and the width correspond to the input size of the LSTM cell and the length of the input sequence, respectively. The topology comprises two input layers that feed images column by column taken from left-to-right and right-to-left along the width dimension *C*. The hidden layer is based on a single Bi-LSTM layer, composed of *forward* and *backward* unidirectional LSTM layers, each comprising a distinct set of $N^{H}=100$ LSTM cells with peephole connections. The two unidirectional layers process the input image from left-to-right and right-to-left, respectively. Each layer generates an output sequence of length *C* and feature size $N^{H}$. Before the dense layer, the *forward* output sequence and the *backward* sequence taken in reverse order are concatenated along the feature dimension. To improve convergence during training, a batch normalization step is applied on the output sequence of the Bi-LSTM layer. The result of the concatenation is fed to a common dense layer, which maps each column of the concatenated output sequence to a vector of $N^{O}=105$ features. Each feature corresponds to a symbol in the alphabet, including a blank space. A Softmax layer converts the output of the dense layer into a vector of probabilities over the alphabet. The values can be positive, negative, zero, or greater than one, but the Softmax transforms them into values between 0 and 1, so that they can be interpreted as probabilities. A CTC layer is used as a loss function. The classification accuracy is based on CER computed as the Levenshtein distance [76] between a decoded sequence and a ground truth.

At inference, the batch normalization layer is merged with the dense layer into an *Output Layer*. We replace the Softmax activation and the CTC with *MaxPerColumn* and *Labeling* functions. The *MaxPerColumn* finds a label with the highest value per column and forwards its index (label) and corresponding value to the next function. The *Labeling* function processes the labels from left to right. A label corresponding to a class zero is a blank space that separates characters and should not be confused with a space between the words. The *Labeling* function continues processing until the next blank and finds a label with the maximum value in the block between the blanks. A label with the maximum value is associated with a character that is considered to be represented between the blanks. After decoding the complete input text line, the function outputs a string of labels.

## 4. *i*DocChip Background

As stated previously, the *i*DocChip system is an FPGA-based end-to-end OCR consisting of layout analysis and character recognition steps of anyOCR (see Figure 1). For each of the four processing steps, FPGA-based systems were introduced and implemented on Xilinx Zynq-7045 SoC [26,27,28,29,30]. In these publications, the performance and energy-efficiency of their implementations were compared to the original Python-based anyOCR software running on Intel Core i7. In this section, the designs and architectures of the four FPGA-based systems are explained briefly.

### 4.1. Binarization

**Figure 4:**
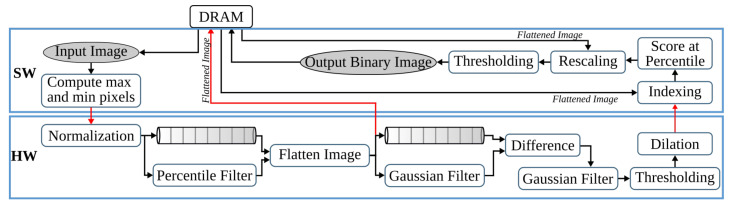
The system-level architecture of the *i*DocChip binarization step.

The first pipeline step of anyOCR converts the input grayscale image into binary using PBB. The hybrid hardware-software FPGA-based accelerator of this anyOCR pipeline step is presented in [26]. To design an efficient heterogeneous architecture of this accelerator, the PBB algorithm is partitioned into hardware and software parts, as shown in Figure 4. The binarization architecture is then implemented in a Zynq-7045 device, where the PL contains the hardware part of the algorithm and PS runs the software. In this implementation, the PL and PS of Zynq can run concurrently while communicating through the advanced extensible interface (AXI) ports. Custom direct memory access (DMA) controllers are implemented to transfer data between PS and PL. The implemented accelerator, running at 166 MHz, outperforms the runtime of the original anyOCR software by 20×. Furthermore, it achieves an energy efficiency at least 70× higher compared to the low-power embedded processors (ARM Cortex-A9, ARM Cortex-A53).

### 4.2. Text and Image Segmentation

**Figure 5:**
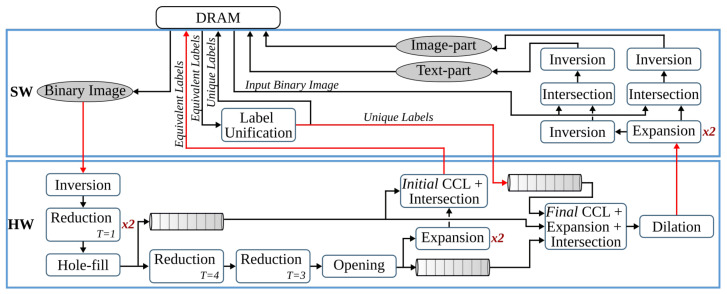
The system-level architecture of the *i*DocChip text and image segmentation step.

This pipeline step separates the text and non-text parts of a document image, using a multiresolution morphology-based text and image segmentation method. In [30], we have presented the first heterogeneous hardware-software architecture based on an optimized version of the text and image segmentation algorithm of anyOCR. Furthermore, this algorithm is partitioned into hardware and software parts, as shown in Figure 5, where time-critical operations with high parallelization capability are offloaded to the FPGA fabric. Based on this architecture, a heterogeneous hardware-software system is implemented on the Zynq-7045 device. Compared to the original anyOCR implementation, this accelerator has achieved a 3.7× speedup in performance and a 139.8× improvement in energy efficiency. Moreover, the hybrid accelerator has outperformed the optimized software implementation of the text and image segmentation algorithm by 2.7× and 127× in terms of performance and energy efficiency, respectively. However, the software part of this hybrid accelerator is very inefficient due to the sequential and time-consuming union-find algorithm. Hence, in [27], we have presented a new optimized heterogeneous architecture based on an improved text and image segmentation algorithm. The resulting accelerator has reduced the runtime and improved the energy efficiency of the previous accelerator stated in [30] by 40% and 46%, respectively.

### 4.3. Text Line Extraction

**Figure 6:**
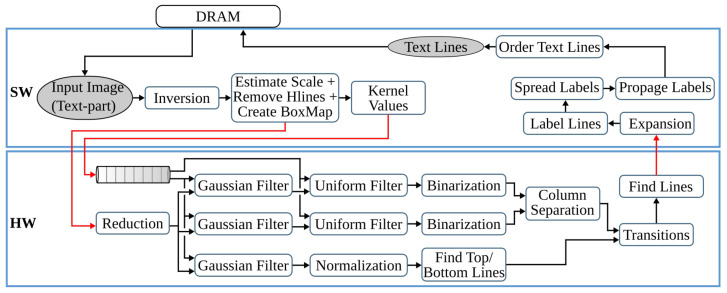
The system-level architecture of the *i*DocChip text line extraction step.

The final preprocessing step extracts the text lines using a Gaussian smoothing-based algorithm. This method has four major processing blocks to estimate text scale, separate columns, find text lines, and extract the lines from the image. The several filters involved in these processing blocks contribute to 85% of the runtime of the text line extraction step. Hence, in [28], the original software algorithm of this anyOCR step is highly optimized and partitioned into hardware and software parts in order to design an efficient heterogeneous architecture, as shown in Figure 6. Based on this architecture, a hybrid accelerator is implemented on a Zynq-7045 device. The resulting hybrid FPGA-based system for the text line extraction step outperforms the original anyOCR software implementation by 135× and 1116× in terms of runtime performance and energy efficiency, respectively.

### 4.4. Text Line Recognition

**Figure 7:**
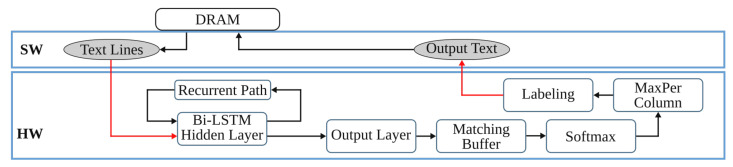
The system-level architecture of the *i*DocChip text line recognition step.

The anyOCR system uses a Bi-LSTM neural network with CTC for text line recognition that allows transcribing the text lines without partitioning them down to separate characters. In [29], we presented the first hardware architecture of the Bi-LSTM network used for OCR. The hardware design is depicted in Figure 7. Most of the steps are based on computing dot products and operations on multiple independent channels that can be efficiently parallelized in hardware. As a result, the complete step is implemented in hardware. The implementation is optimized for online processing, i.e., no batch processing, meaning that the text lines are processed one after another as soon as they are available from the last preprocessing step. Compared to the original anyOCR software running on i7-4790T, the accelerator provides 71× speedup and 935× higher energy efficiency. Meanwhile, it achieves at least 67× higher energy efficiency than embedded processors.

### 4.5. The anyOCR System vs. Separate *i*DocChip Components

**Figure 8:**
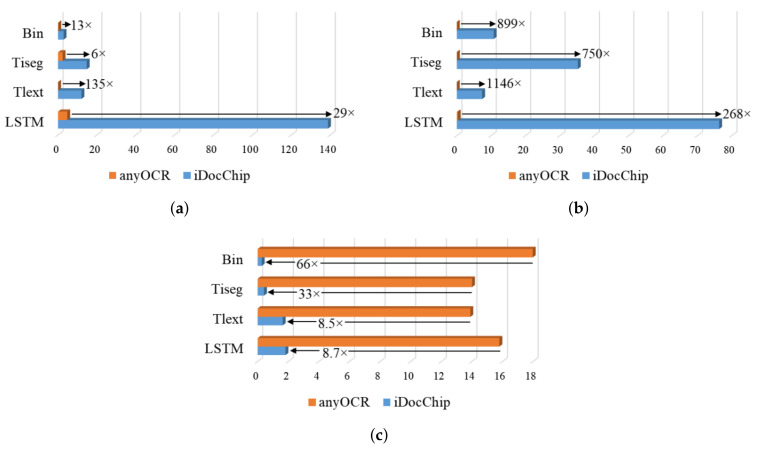
Comparison of anyOCR and iDocChip pipeline steps in terms of (**a**) throughput (FPS), (**b**) energy efficiency (FPS/W), (**c**) power consumption (W). *Bin*, *Tiseg*, *Tlext*, *LSTM* stand for binarization, text and image segmentation, text line extraction, and Bi-LSTM-based text line recognition, respectively.

Compared to anyOCR the throughput and energy efficiency are increased by a minimum of 6× and 268×, while power is reduced by 8.5×, as shown in Figure 8.

## 5. Algorithmic Optimizations and Hardware-Software Partitioning for the End-to-End *i*DocChip

The anyOCR chain comprises various algorithms with a different potential for parallelism. Some algorithms are sequential and prevailed with control-flow, while others are dominated by the dataflow and highly parallelizable. As a result, efficient hardware-software partitioning becomes crucial for designing an accelerator that meets the real-time processing requirements at low-power consumption.

### 5.1. Binarization

**Figure 9:**
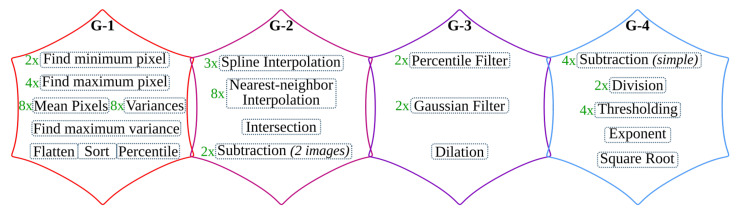
Operations involved in binarization processing pipeline grouped from *G-1* to *G-4*.

The overall operations involved in the binarization processing pipeline are given in Figure 9 and broadly categorized into four groups. The operations grouped in G-1 are sequential computations that do not benefit from hardware parallelism. The *G-2* operations can be offloaded to hardware; however, they are highly resource-intensive. Operations categorized in the *G-3* group are window-based computations that have high parallelization capability. The simple arithmetic in *G-4* are hardware-friendly and hence, they are the most suitable to implement on the FPGA fabric.

In the previous *i*DocChip publication for binarization processing pipeline [26], as input images were obtained by scanning the document pages, they are less likely to be skewed. Hence, as shown in Figure 4, the previous work avoids the *skew angle estimation* and *image rotation* operations involved in the original anyOCR algorithm. However, these algorithmic adjustments have caused a reduction of accuracy from 76.3% to 75.92% for the test dataset [25]. Furthermore, due to other modifications of the binarization algorithm, the previously mentioned work has a 75.4% recognition accuracy for the given dataset. However, as the new generic *i*DocChip design is not limited to scanned images, it includes the *skew angle estimation* and *image rotation* operations. Our system compensates skewed documents by up to 5 degrees to enable OCR for images taken by a hand-held camera.

#### 5.1.1. Binarization: *G-1* Operations

*Normalization*, *skew angle estimation*, and *image rotation* operations involve calculations of minimum ($P_{min}$) and maximum ($P_{max}$) pixels. These calculations, including mean pixel, variance, and maximum variance computations required by the *image rotation* block and the sort operation of the *score at percentile* operation, are sequential as they require traversing all pixels of an image. In order to accelerate these operations, it would require comparing multiple values in parallel. However, as large sorting networks are resource-demanding, it becomes more efficient to perform sorting in software.

**Figure 10:**
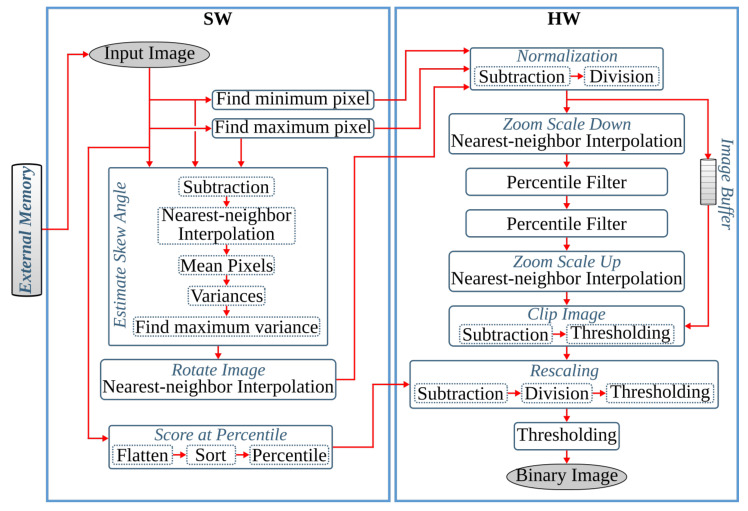
Hardware-software partitioning of the binarization process for the *i*DocChip system.

Moreover, to limit data transfer between the FPGA fabric and CPU, the *skew angle estimation* and *image rotation* operations are computed before the image is streamed from the CPU. Hence, they are relocated to the beginning of the binarization process and work on the original input image. As a result, the maximum and minimum pixels computed for the normalization process are also used to calculate the skew angle. Additionally, the *cubic spline interpolation* of the *rotate image* operation is replaced by the simple and fast *nearest-neighbor interpolation*. The resulting rotated image is then transferred from the CPU to the hardware fabric to initiate the binarization process of the *i*DocChip system (see Figure 10). In our experiment, these two adjustments positively affect recognition accuracy when tested on our dataset [25], as shown in modification type *BIN-1* and *BIN-2* of Table 2.

The high- hs and low-score ls pixel values are not severely manipulated by the operations prior to the *score at percentile* operation (see Figure 2a). Hence, the *score at percentile* operation is processed in software using the original input image. The computed hs and ls parameters are then transferred to the hardware to compute the rest of the binarization output, as shown in Figure 10. The relocation of *score* calculations has a negligible accuracy loss; see type *BIN-3* of Table 2. Relocating the *score at percentile* operation not only optimizes the hardware implementation but also eliminates the need for calculating a mask image; refer to Figure 2a and Figure 10. Removing the *compute mask* block saves very large resources that would have otherwise been used to implement (1) two *Gaussian* and one dilation morphological filters that have very large kernel sizes, (2) the inter-image *subtraction* and *intersection* operations that require the rotated clip image, $I_{rclip}$, to either be stored within the hardware logic cells or in an external memory until the corresponding *Gaussian filter* and *dilation* results are available, and (3) the *exponent* and *square root* operations.

#### 5.1.2. Binarization: *G-2* Operations

The zoom operations of the binarization processing pipeline require two *cubic spline interpolation* tasks, which are resource-intensive to implement in hardware. Down-scaling the image using *zoom-out interpolation* (refer Figure 2a) relieves the follow-up *percentile* operations from unnecessary computations and reduces their runtime. In the new *i*DocChip system, the *zoom interpolation* operations are implemented in hardware. However, these operations are replaced by the hardware-friendly *nearest-neighbor interpolation*. When this modification is applied to our test dataset, it has resulted in a slight shift in accuracy; see Table 2, modification types *BIN-4*. Moreover, to further reduce the number of required computations for the percentile filters, the *zoom scale down* (as well as the *zoom scale-up*) values are increased. For example, increasing the zoom factor from 3 to 8 does not significantly affect the system’s accuracy, see Table 2, modification types *BIN-5*.

The *clip image* operation used to *estimate background* calculates the difference between the result of the second *percentile filter*, $I_{perc}$, from the corresponding pixels of the normalized image $I_{norm}$, as explained in Section 3. Since the zoom and percentile operations are relatively fast, a limited number of pixels from the $I_{norm}$ are buffered using on-chip storage units until the pixels from $I_{perc}$ are ready. As described in Section 5.1.1, the rest of the operations in *G-2* are removed.

#### 5.1.3. Binarization: *G-3* Operations

The percentile filters process on a highly scaled-down image; as a result, their kernel sizes are appropriately adjusted. For example, for our test dataset, the kernel sizes of the *percentile filters* are changed from 20×3 and 3×20 to 7×1 and 1×7, respectively. Moreover, to process these operations in a hardware-friendly manner, the order of their operation is interchanged, where the horizontal *percentile filter* with KS=1×7 is processed ahead of the vertical *percentile filter* with KS=7×1, see details in Section 6. This change in the order of operations has only a minimal accuracy loss; see modification type *BIN-6* of Table 2. The other window-based operations, *Gaussian* and *dilation* filters, are removed as explained in Section 5.1.1.

#### 5.1.4. Binarization: *G-4* Operations

As described in Section 5.1.1, the *compute mask* block is removed. Hence, the *exponent* and *square root* operations are not implemented. The rest of the operations in the *G-4* category are basic arithmetic operations with low resource utilization that are easily parallelizable.

After the algorithmic modifications and mapping of operations into the suitable computing platforms, the resulting hardware-software partitioning of the binarization algorithm for the *i*DocChip system is summarized in Figure 10.

### 5.2. Text and Image Segmentation

**Figure 11:**
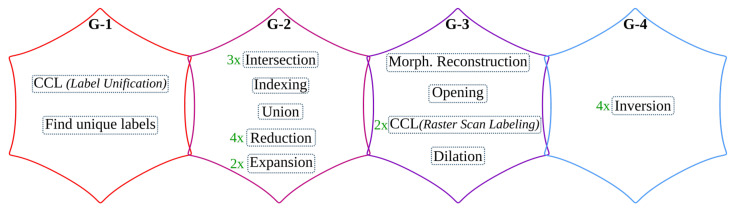
Operations of the text and image segmentation processing pipeline grouped from *G-1* to *G-4*.

Similar to the binarization processing pipeline, the operations in the text and image segmentation step are categorized into four groups, as shown in Figure 11. The accuracy shift caused by the algorithmic adjustments is also tested using the sample test dataset [25].

#### 5.2.1. Text and Image Segmentation: *G-1* Operations

Connected component labeling (CCL) is a window-based operation used to label connected components of an image. The classical CCL operation involves three tasks: *initial labeling*, *label unification*, and *final labeling*. The first and last tasks operate by sliding the computational window on the input image in a raster scan manner. These window-based tasks of CCL are suitable to directly implement on hardware. However, *label unification* is a sequential task that does not benefit from hardware parallelism. A CCL operation followed by *feature extraction*, collectively known as connected component analysis (CCA), measures and analyzes features of component regions and executes certain decisions. As shown in Figure 2b, the *feature extraction* operation of text and image segmentation algorithm uses two images to *find unique labels* and generate output through union and indexing.

**Figure 12:**
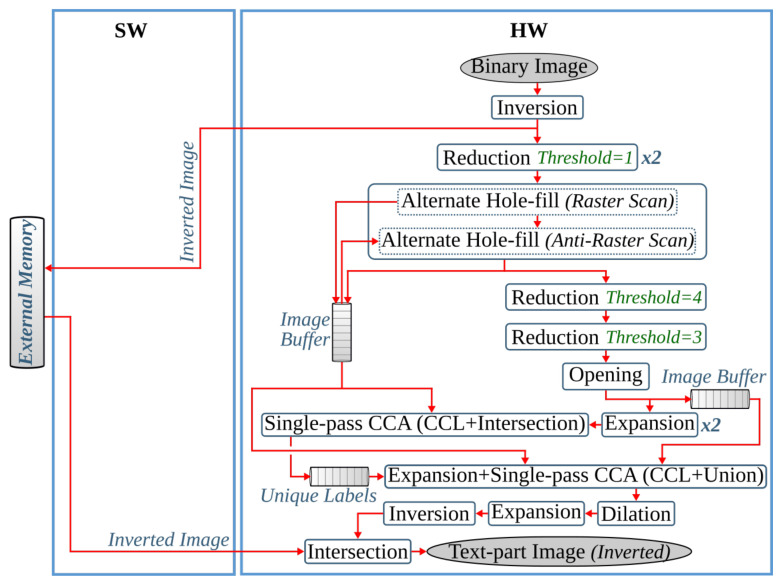
Hardware-software partitioning of text and image segmentation for the *i*DocChip system.

In the literature [77,78,79,80,81,82], there exist several CCAs algorithms, such as the *single-pass CCA*, that are specifically optimized for hardware implementations. In a single-pass CCA, the required features of connected components are extracted together with the first raster scan used to label the image (*initial labeling*). This algorithm bypasses *label unification* and *final labeling* tasks of CCL; hence it does not output a labeled image. However, the *feature extraction* operation of the text and image segmentation step requires the complete labeled image to extract unique labels. As a result, we apply two single-pass CCA operations to avoid the separate *label unification* process. Here, the second raster scan can be processed only after the complete processing of the first raster scan. Moreover, the mask and seed images are required for both computations. Since the seed and mask images are already scaled down into smaller sizes, these images are stored within on-chip buffers to avoid several data transfers to/from the CPU. To further speed up the performance of the system, *expansion* and the operations involved for *feature extraction* (*intersection*, *find unique labels*, *indexing*, and *union*) are overlapped with the single-pass CCAs (see Figure 12). These hardware-specific optimizations do not affect the overall accuracy of the system.

#### 5.2.2. Text and Image Segmentation: *G-2* Operations

The *image-part* result of the text and image segmentation step is not processed further; refer to Figure 2b. Hence, for the end-to-end *i*DocChip system, it is not computed. As a result, one *intersection* and *inversion* operation are eliminated from the text and image segmentation pipeline. However, the inverted binary image $I_{inv}$ is still required to compute the *text-part* output ($I_{tp\_mask}$). In order to avoid buffering the complete image within the limited block random-access memory (BRAM) resources of the FPGA, $I_{inv}$ is stored in the external memory. This image is streamed back to the hardware when the *intersection* operations are ready to be computed; see Figure 12.

As described in Section 5.2.1, the CCL and *feature extraction* operations require the mask $I_{mask}$ and seed $I_{seed}$ images for the *intersection* and *union* operations. As these images are scaled down by a factor of four, they have a lower resource overhead. Hence, they are buffered using on-chip storage units.

The *reduction* and *expansion* operations are implemented in hardware. However, these operations generate result pixels irregularly that challenge the streaming architecture of the hardware. For a *reduction* operation, a 2×2 block of four pixels is required to produce a result. Hence, it outputs pixels for every second row of the incoming pixels. On the other hand, the *expansion* operation populates a pixel into a 2×2 block of four pixels. The streaming irregularity of these operations is hidden by manipulating the *expansion* operation and using on-chip buffers; refer to Section 6.1.3.

#### 5.2.3. Text and Image Segmentation: *G-3* Operations

The *morphological reconstruction*-based hole-filling algorithm uses a reconstructive *erosion* operation to fill the holes within an image. However, as this computation is an iterative process, it requires a large number of raster scans. For instance, for the test dataset images given in [25], the number of scans needed only for a 4-connective window-based *hole-fill* operation range from 198 to 827. Hence, *morphological reconstruction* is not suitable for a streaming-based dataflow hardware architecture. To overcome this issue, an alternative custom hole-fill algorithm is used, as explained in detail in [27]. This operation achieves the same results as the *morphological reconstruction by erosion* in fewer iterations. Moreover, with a slight accuracy loss, the *alternate hole-fill* algorithm is able to accomplish the task with only two iterations; see modification type *TISEG-1* of Table 2.

Hence, the *i*DocChip system uses the *alternate hole-fill* algorithm with a 4-connective SE, with only two iterations. After processing the initial hole-fill operation in a raster scan manner, the result is processed in an anti-raster scan direction (from bottom-right to top-left). To run the second sequence in an anti-raster scan manner, the first iteration must finish processing the complete image. As a result, after the initial raster scan operation, the resulting image is stored within the hardware to avoid data transfer to external memory. These on-chip buffer units are also re-used to store the resulting mask image of the *alternate hole-fill* operation $I_{mask}$ since it is required for the *intersection* operation in *feature extraction*, as explained in Section 5.2.2. For implementation details; refer to Section 6.1.2.

The window-based raster scan labeling operations of the single-pass CCA are implemented in hardware as per the description given in Section 5.2.1. The *opening* and *dilation* morphological operations are also implemented in hardware as they are highly parallelizable.

#### 5.2.4. Text and Image Segmentation: *G-4* Operations

As explained in Section 5.2.2, the *inversion* operation used to extract the *image-part* of the document is not required; hence it is omitted. Moreover, the last inversion operation is not needed, as the next processing pipeline (text line extraction) works on the inverted image of the document’s text-part. The remaining two *inversion* operations are implemented in hardware.

### 5.3. Text Line Extraction

**Figure 13:**
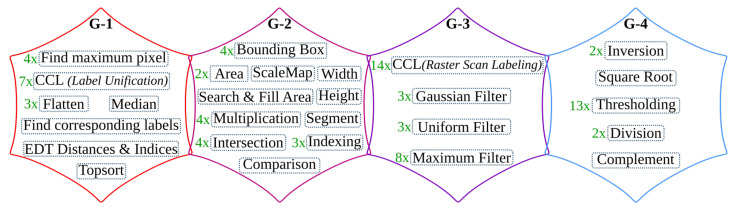
Operations involved in text line extraction processing pipeline grouped from *G-1* to *G-4*.

As shown in Figure 13, the overall operations involved in the text line extraction processing pipeline are broadly categorized into four groups. Similar to Figure 9 and Figure 11, these four categories are also grouped from left to right with respect to suitability for hardware implementation. The most left *G-1* groups of operations are sequential, while the most right *G-4* operations are highly parallelizable.

#### 5.3.1. Text Line Extraction: *G-1* Operations

As mentioned in Section 5.1.1, the calculation of maximum pixels is not suitable for hardware implementation. Hence, to avoid the four maximum pixel computations required in the text line extraction step, these values are replaced by fixed thresholds. As a result, the thresholding computations in *smooth text region*, *find column edges*, *top and bottom edge computations* use constant threshold values instead of computing maximum pixel values. These values are transferred directly from the CPU. When applied to our test dataset, these adjustments have only reduced the overall accuracy by 1%; see Table 2, modification type *TLEXT-1*.

As explained in Section 3.3, a topological sort algorithm is used to arrange the extracted text lines in reading order. For this task, however, a simple, quick sort operation works more efficiently than the topological sorting algorithm due to the regularity of the order of text lines. The increase in accuracy due to this algorithmic optimization is shown in modification type *TLEXT-2* of Table 2.

**Figure 14:**
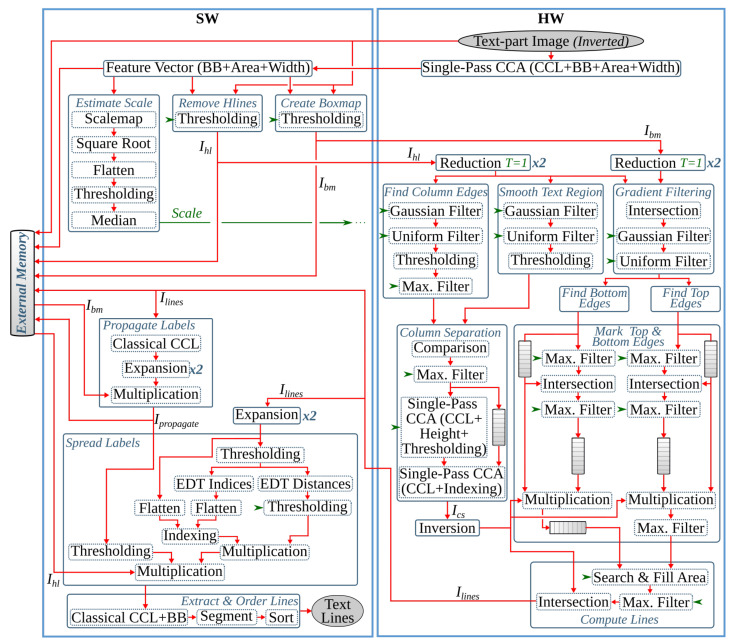
Hardware-software partitioning of text line extraction for the *i*DocChip system.

As described in Section 5.2.1, a classical CCL requires two raster scan computations and a label unification process. AS anyOCR uses classical CCL, it requires a total of 14 raster scan labeling and 7 label unification tasks for the text line extraction step. Similar to the CCL in the text and image segmentation step, these operations are replaced by the hardware-friendly single-pass CCA to design the *i*DocChip system. Moreover, different CCA operations that work on the same image are combined to speed up the system. Hence, the CCL operations required for *estimate scale*, *find and remove horizontal lines*, and *create boxmap* are combined, as shown in Figure 14.

The next CCL operation is used in the *column separation* sub-block to select the relevant column lines. Similar to the other CCA operations, the *feature extraction* operation of *column separation* correctly identifies the long connected components only at the end of the first single-pass CCA; therefore, in *i*DocChip, the single-pass CCA is applied twice for the *column separation* sub-block; refer to Section 6.1.2 for details. The intermediate images of this block are stored using on-chip memory units, as the image has been downscaled by two *reduction* operations; see Figure 14.

The *label propagation* operation also involves two CCL tasks. The first one labels the connected components of the boxmap image $I_{bm}$, while the second CCL labels the image $I_{lines}$ that contains the detected lines of the document image. Then the corresponding labels of these two images are identified and they are used to index the labeled lines, as described in Section 3.3. In *i*DocChip system, however, the label propagation operation is optimized in such a way that the labeled $I_{lines}$ image is directly multiplied by the binary boxmap image $I_{bm}$. This optimization eliminates one CCL task and replaces the *feature extraction* operations (*find corresponding labels* and *indexing*) with a single *multiplication* operation without highly affecting the system accuracy, see modification type *TLEXT-3* of Table 2. To avoid memory bandwidth congestion with multiple data transfers between the CPU and the FPGA fabric, the *label propagation* operation is mapped to software and uses classical CCL; see Figure 14.

The last CCL task is used in the *extract & order lines* operation to label the text lines and find their bounding boxes. This task is followed by *segmentation* and *sorting*. Due to the sequential nature of the *sorting* algorithm, it is best suited for software-based implementation. As a result, the three operations of *extract & order lines*, including the classical CCL, are mapped to software in order to limit the number of data transfers between the software and hardware-based platforms.

As detailed in Section 3.1, to compute the final value of the *estimate scale*, a *ScaleMap* array is constructed, where each pixel of a connected component is replaced by the square root of the area of its component. The resulting two-dimensional array contains very large data that is not desirable to store using on-chip memory. Moreover, the *ScaleMap* is flattened into a one-dimensional array in the next steps to apply thresholding and find the median value. These tasks are also sequential, as they require sorting of a 2D array of imageheight*imagewidth. As a result, after the single-pass CCA, consequent steps of the *estimate scale* are processed in the CPU.

The *find and remove horizontal lines* and *create boxmap* operations require the labeled image and the computed *Scale* value; refer to Figure 2c. Hardware implementation of these steps is possible (1) by transferring the labeled data from the external memory to hardware, which requires high memory bandwidth or (2) by transferring the binary image from the external memory to hardware and recomputing the single-pass CCA, which is also unnecessary, as the labeled image is already stored in the off-chip memory. For these reasons and to allow a different memory access pattern for the upcoming *reduction* operation, the *find and remove horizontal lines* and *create boxmap* are computed on CPU. The resulting $I_{hl}$ and $I_{bm}$ images are then transferred to hardware (see Figure 14). These hardware-specific optimizations of CCL and *feature extraction* operations do not affect the system accuracy.

Furthermore, the *Euclidean distance transform* and *flattening* of two-dimensional images of the *spread labels* block are sequential operations that do not benefit from hardware parallelization. As a result, similar to *estimate scale*, the complete *spread labels* operations are mapped to software; see Figure 14.

#### 5.3.2. Text Line Extraction: *G-2* Operations

As described above, due to the combination of multiple CCA tasks, the number of *bounding box* operations is reduced from four to two, one at the beginning of the text line extraction step and the other to *extract & order lines*. Similarly, the number of *indexing* operations is also reduced from three to two due to the modification of *label propagation* operation. The *indexing* required for *column separation* is implemented in hardware while the other is mapped to software. Except for the *ScaleMap* computation and the text line *segmentation*, the rest of *G-2* operations are realized in hardware.

#### 5.3.3. Text Line Extraction: *G-3* Operations

The operations categorized in *G-3* are window-based computations that have high parallelization capability. However, the large window sizes of *Gaussian*, *uniform*, and *maximum* filters require a massive number of line buffers when implemented in hardware. A *Gaussian filter* is achieved by convolving the 2D Gaussian distribution function with the image, while *uniform* and *maximum* filters compute the average and largest values, respectively, from a given window. In the text line extraction processing pipeline of anyOCR, the kernel sizes of the *Gaussian*, *uniform* and *maximum* filters are dependent on the previously estimated *Scale* value of the text size. For example, for our test dataset given in [25], the smallest estimated *Scale* value is 33. Hence, for the *Gaussian*, *uniform*, and *maximum* filters, the system requires minimum kernel sizes of values (265×132), (330×1), and (33×165), respectively. Hardware implementation of these filters with such large parameters is not feasible. Therefore, for the *i*DocChip system, we have introduced two *reduction* operations of T=1 before applying the window-based computations, as shown in Figure 14. The *Scale* value is also adjusted appropriately, which reduces the kernel sizes of the filters. After computing the text lines, the image is scaled back up to its original using *expansion* operations. These optimizations have a positive effect on the system’s overall accuracy as shown in *modification type TLEXT-4* of Table 2 for our test dataset [25].

As described in Section 5.3.1, in the *i*DocChip system, five classical CCL operations that require ten *raster scan labeling* and five *unification* tasks are replaced by three single-pass CCAs, and they are implemented in hardware. The other two classical CCL operations run on the CPU.

#### 5.3.4. Text Line Extraction: *G-4* Operations

As the operations categorized in *G-4* are hardware-friendly, they are realized in hardware (except for those involved in *estimate scale*, *remove Hlines*, *create boxmap*, *propagate labels*, and *spread labels*).

### 5.4. Text Line Recognition

**Figure 15:**
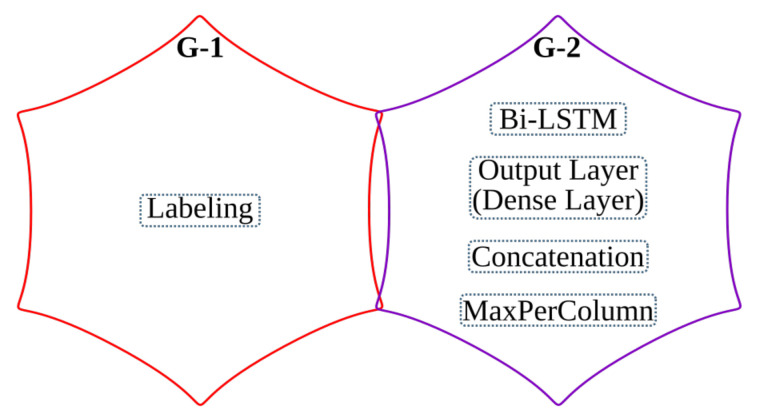
Operations of the text line recognition processing pipeline grouped from *G-1* to *G-2*.

As shown in Figure 15, the overall operations involved in the text line recognition processing pipeline are broadly categorized into two groups. The *G-1* operations are sequential, while the *G-2* operations are parallelizable and benefit from the hardware implementation.

#### 5.4.1. Text Line Recognition: *G-2* Operations

The topology used for text line recognition comprises functions with various potential for parallelism. The Bi-LSTM has restrained parallelism due to recurrent connections. The next step, the image column in the case of OCR, can be processed if the previous step has been finished due to a precedence constraint that restrains the parallelism compared to feed-forward neural networks. However, Bi-LSTM maintains multiple other levels of parallelism. A coarse-grained output parallelization can be applied on LSTM cells and fine-grained input parallelization on LSTM’s gates and dot products. The LSTM’s available parallelism is sufficient for a real-time inference of OCR and similar tasks on embedded platforms. The dense layer that is a feed-forward neural network maintains coarse-grained output parallelism applied on a level of neurons and fine-grained input parallelism on dot products. However, as it follows the Bi-LSTM layer, the parallelism cannot span over several columns. The following operations, *Concatenation* and *MaxPerColumn*, maintain output parallelism of the presiding layers. All functions mentioned above benefit from hardware implementation, as FPGAs enables multiple levels of parallelism with small or no synchronization overhead.

#### 5.4.2. Text Line Recognition: *G-1* Operations

In contrast, the *Labeling* function is inherently sequential. As explained in Section 3.4, the function does not maintain any parallelism, as the columns (labels with corresponding probability) can be processed only one after another. However, there is a strong reasoning for implementing it in the hardware. The hardware-software partitioning should consider not only the potential for parallelism but also the cost of memory transfers from hardware to software and vice versa. The *Labeling* function operates on a small buffer with a size proportional to the number of characters in a text line that is typically order of magnitude smaller than the number of columns in an image. The hardware implementation cost of such buffer is overrun by the overhead due to data transfers to the external memory of outputs from the *MaxPerColumn* with the size proportional to the number of columns.

## 6. *i*DocChip Hardware Architecture

To design the *i*DocChip system, the preprocessing steps of anyOCR are optimized, and a hardware-software partitioning is applied as described in the previous section. Most of these operations are offloaded to the FPGA and implemented as hardware blocks (IP cores). On the FPGA, the incoming pixels are streamed row by row in a raster scan order (top-to-bottom, left-to-right). The *i*DocChip adopts pipelined dataflow architectures.

### 6.1. Hardware Architectures of Preprocessing Operations

**Figure 16:**
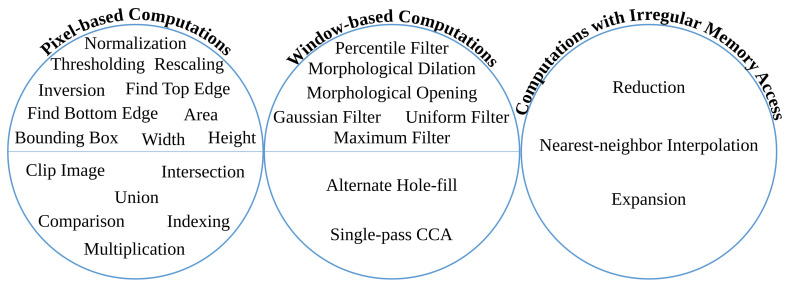
The system-level architecture of the *i*DocChip binarization step.

As shown in Figure 16, the preprocessing operations that are mapped into the hardware can be broadly classified into three groups: *pixel-based computations*, *window-based computations*, and *computations with irregular memory access*.

#### 6.1.1. Pixel-Based Computations

Operations categorized in this group involve simple mathematical calculations. The top half operations of the *pixel-based computations* shown in Figure 16 require only a single image to generate a result (i.e., single-image pixel-based operations). In contrast, the bottom half work on multiple images to produce an output (i.e., multiple-image pixel-based operations). Given all the required inputs, *pixel-based computations* require only a single clock cycle to process a pixel. Moreover, they are highly parallelizable, as image pixels are computed independently from their neighborhood. Such operations can even process several pixels per clock cycle by adjusting the number of input pixels.

*Normalization* is the first step in the binarization processing pipeline. As shown in Equation (1), to calculate a normalized pixel $P_{norm}$, the minimum $P_{min}$ and maximum $P_{max}$ pixel values of the image are required. As described in Section 5.1.1, these values are computed on the CPU. The resulting $P_{min}$ and $P_{max}$ are sent to the hardware fabric along with the pixels of the original grayscale input image. The $P_{min}$ and $P_{max}$ values and also the input pixels are represented in hardware using 8-bit unsigned integers. The normalization process results in 5-bit fixed point normalized $P_{norm}$ pixel values.

In the binarization and text line extraction steps, several *thresholding* operations are used in order to select target pixels or components. As described in Section 5, about half of the *thresholding* computations are realized in hardware and implemented as a simple comparison logic. To allow a wide range of applications, the *threshold values* required to compute the results are passed directly from the CPU. In the hardware, these values are represented as fixed-point numbers. Depending on the underlying task, *thresholding* outputs binary pixels or fixed point results.

The *rescaling* operation of the binarization step applies thresholding on the incoming image using the equation given in Equation (3). As described in Section 5.1.1, the low ls and high hs score values used in the *rescaling* operation are calculated from the original grayscale image in the CPU. These parameters are then transferred to the hardware, where they are stored as 8-bit unsigned integers. After applying the *rescaling* operation, the resulting 8-bit pixels are streamed to the following processing block.

To *find the top and bottom edges*, the optimized algorithm for *i*DocChip uses a modified equation Equation (7) as opposed to the original Equation (6) from the anyOCR system. As a result, the hardware block of this optimized operation only requires two’s complement, division, and thresholding.

(7) $$I_{top}=\left\{\begin{array}{cc} I_{grad}/T_{1}, & I_{grad}>0\\ 0, & otherwise \end{array}\right.\;\;I_{bottom}=\left\{\begin{array}{cc} -I_{grad}/T_{2}, & I_{grad}<0\\ 0, & otherwise \end{array}\right.$$

In the original anyOCR software, the text and image segmentation step outputs an inverted image that is inverted back in the text line extraction step. However, in the end-to-end OCR system, intermediate results of the pipeline steps are not required. Therefore, the *i*DocChip system has only one *inversion* operation at the beginning of the text and image segmentation processing pipeline. This *inversion* operation is implemented in hardware by complementing the binary incoming pixels.

The feature vectors (*area*, *bounding box*, *width*, and *height*) mapped to hardware are calculated during the single-pass CCA operations of the text line extraction step. Although connected components may have an irregular structure, they are assumed to be enclosed in a rectangular shape for the *bounding box* computations. Hence, for operations that require *bounding box*, i.e., *estimate scale* and *extract & order lines*, four coordinates of connected components ($X_{1}$, $X_{2}$, $Y_{1}$, and $Y_{2}$) are tracked and updated. The *area* of connected components is calculated from the *bounding box*. However, only the *X* or *Y* coordinates suffice to compute the *width* or *height* of connected components, respectively.

*Multiple-image pixel-based operations* take one pixel from each input image to generate a result pixel. Therefore, they require corresponding pixels of all input images to be readily available. The hardware accelerator of *i*DocChip has a stream-based dataflow architecture. Therefore, to avoid data loss during the processing of the *multiple-image pixel-based operations*, sufficient on-chip buffers are utilized to store pixels of the faster processing line, enabling these operations to achieve a throughput of one pixel per clock cycle. As shown in Figure 10, Figure 12 and Figure 14, multiple image buffers are used to synchronize the pixel streams for *clip image*, *intersection*, *multiplication*, *union*, *comparison*, and *indexing* operations.

#### 6.1.2. Window-Based Computations

**Figure 17:**
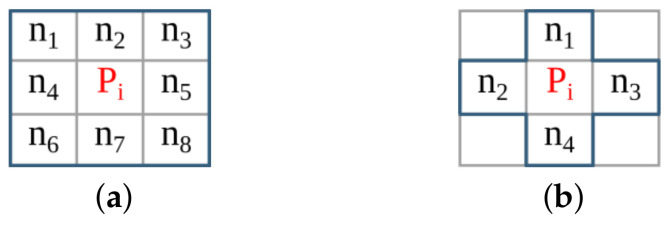
A 3×3 sliding window (**a**) an 8-connective (square) kernel (**b**) a 4-connective (cross) kernel.

Image filtering, feature detection, and tracking operations often use a *sliding window*, also known as a *kernel* or a *structuring element (SE)*, to process pixels and output results. For each pixel $P_{i}$ centered at a neighborhood *N*, the *window-based operations* calculate the neighboring pixel values by employing a given operator. Depending on the task/filter, different neighborhood operators compute different functions, such as convolution, sorting, averaging, comparison, and others. There are two basic types of windows: square-connective and cross-connective, as shown in Figure 17.

Many *window-based computations*, such as morphological operations, Gaussian, uniform, and maximum filters, share a feature called *separability*, which allows kernel decomposition. As a result, the kernels of these operations that utilize a rectangular window can be separated into two one-dimensional windows. For example, a 3×3 Gaussian filter can be computed by applying the convolution operator using a 1×3*horizontal window* and a 3×1*vertical window*. The *separability* feature allows for fast computation, such that when a filter of kernel size $\left(k_{v}\times k_{h}\right)$ is applied on an image of size H × W, it reduces the computational costs of the filter from $O(H\cdot W\cdot k_{v}\cdot k_{h})$ to $O(H\cdot W\cdot \left(k_{v}+k_{h}\right))$.

Serial In-Parallel Out (SIPO) shift registers are used to store neighboring pixels for a horizontal filter and are updated every clock cycle. Before processing a pixel, the horizontal computational window is first prepared by shifting the register values to remove the oldest and insert the current pixel. Contrary to the horizontal filter, the neighboring pixels of a vertical filter are accessed column-wise. To avoid the memory bandwidth congestion that results from reading all input pixels of the window in parallel, the required neighboring pixels of the vertical filter are stored in dedicated *line/row buffers* within the FPGA. For a vertical filter of a kernel size $\left(k_{v}\times 1\right)$, a total of $k_{v}-1$ line buffers are required. These buffers are implemented using BRAMs. Similar to the horizontal filter, after the result of the current pixel is computed, the oldest pixel is removed, and the current pixel is stored into the buffer.

**Figure 18:**
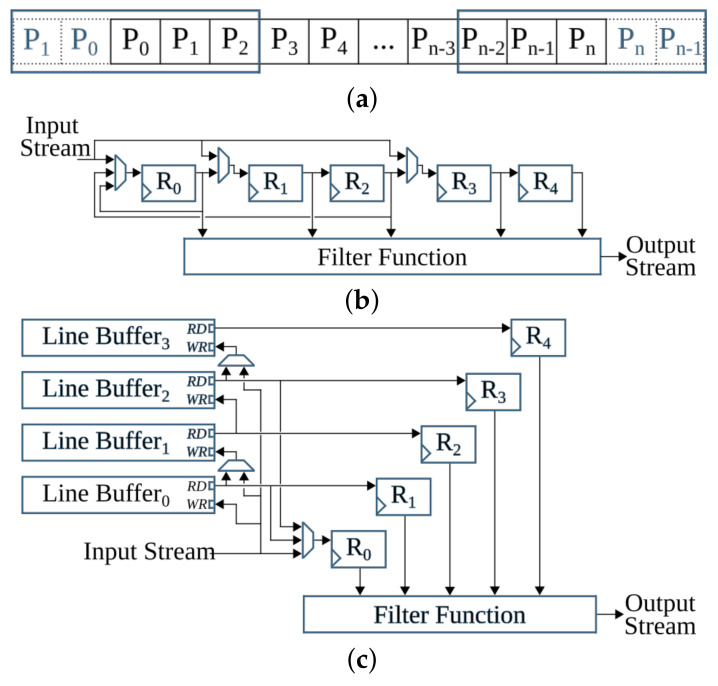
Border handling for a 5×5 separable filter (**a**) reflection of border pixels (**b**) SIPO shift registers for horizontal computation with border reflection logic (**c**) line buffers and window for vertical computation with border reflection logic.

*Window-based computations* require a specific *border handling* strategy when computing results for pixels at/around the image’s border, where the pixels do not have enough neighbors to fill the sliding window. This task extends the image size with respect to the window size such that when a filter with a kernel size of $\left(k_{v}\times k_{h}\right)$ is applied on an image with a height *H* and width *W*, the *border handling* task extends the image height and width to $H+k_{v}-1$ and $W+k_{h}-1$, respectively. The pixel values of the extended image are extrapolated from the border pixels of the input image. There are different *border handling* schemes, such as *reflection*, *mirroring*, *nearest*, *wrap*, *constant*, and others. Border handling techniques require extra logic in hardware implementations of filters. For our implementations of the *window-based operations*, we reflect border pixels to extend the incoming image; see Figure 18a. The neighboring pixels of the computational window at/around border pixels are reflected from the corresponding shift registers or line buffers using multiplexers, as shown in Figure 18b,c.

*Rank-order filters*, like *percentile and maximum filters*, are non-linear filters that sort grayscale pixels of a sliding window and select the pixel located at the given percentile value of the sorted window to generate a result. For the classical *median* and *maximum* filters, the filtered values are taken at the 50th and 100th percentile of the ordered sliding window. In the binarization processing pipeline, there exist two consecutive *percentile* operations. Due to the algorithmic optimization detailed in Section 5.1.3, the two *percentile filters* characterize the separability feature as they involve a horizontal followed by a vertical computation. Hence, to implement these operations, shift registers and line buffers are used to store neighboring pixels for the horizontal and vertical filters, respectively. The border handling strategy is also implemented in a similar manner as shown in Figure 18. Furthermore, the text line extraction step contains eight *maximum filters*. However, due to the *reduction* operation, the kernel sizes of these filters are smaller and manageable. A two-dimensional maximum filter, unlike other rank-order filters, is separable into one-dimensional horizontal and vertical filters. Hence, shift registers and line buffers are used to buffer neighborhood pixels.

*Morphological operations* are also non-linear image processing and analysis techniques that are used to analyze and process an image based on the characteristics of its shape. Two fundamental processes, i.e., *erosion* and *dilation*, are the basis for all morphological operations. These operations compare the neighborhood pixels within the sliding window and set the minimum pixel (for *erosion*) or the maximum pixel (*dilation*). A *morphological opening* operation involves an *erosion* process followed by a *dilation*. The text and image segmentation pipeline of *i*DocChip contains a *dilation* and an *opening* operations with a square- and cross-connective SEs, respectively. The fundamental processing blocks of *morphological operation* are implemented based on [83]. These highly parallelizable hardware blocks are parameterizable to any kernel size. Moreover, due to the *additive* characteristic of *morphological operations*, multiple smaller size processing blocks are concatenated to build larger kernel size filters to provide further flexibility. Due to the *separability* of *morphological operations*, their processing blocks compute results using two consecutive one-dimensional windows and utilize shift registers and line buffers to store neighborhood pixels for horizontal and vertical windows, respectively.

*Gaussian filter* is a separable linear filter that performs a convolution operation using a Gaussian kernel with a standard deviation σ. The text line extraction step of *i*DocChip involves three *Gaussian* operations. In general, the convolution kernel of *Gaussian* is dependent on the purpose of the given task, such as smoothing (e.g., *smooth text region*), edge detection (e.g., *find column edges*), edge enhancement (e.g., *gradient filtering*), etc. For *i*DocChip, the kernel sizes of these *Gaussian filters* are dependent on the estimated text Scale. Hence, the kernel values of these *Gaussian filters* are calculated after computing the *estimate scale* block. Then they are transferred to the hardware and stored as 7-bit fixed-point values. Due to the *separability* characteristic of *Gaussian*, its hardware block computes convolution as a horizontal filter followed by a vertical filter. It uses shift registers and line buffers to store neighborhood pixels for column and row processing, respectively. As these Gaussian filters are computed in parallel, they share the line buffers to reduce BRAM overhead.

A *uniform filter* (also called *mean* or *average filter*) is a linear filter that calculates the mean value of the neighborhood pixels within the sliding window. The text line extraction pipeline of *i*DocChip uses three *uniform filters* with different kernel sizes that are dependent on the *estimate scale* value. Similar to the previously mentioned filters, shift registers and line buffers are used to store neighborhood pixels. To reduce the number of computations required to process a result, a *moving average* is used, where a new pixel result is calculated by computing an average for the new pixel, adding it to the previous pixel result, and removing the average of the oldest pixel. To realize the moving average-based *uniform filters* in hardware, in addition to the shift registers and line buffers used to store neighborhood pixels, an extra register and line buffer are required to store results for the next computation.

**Figure 19:**
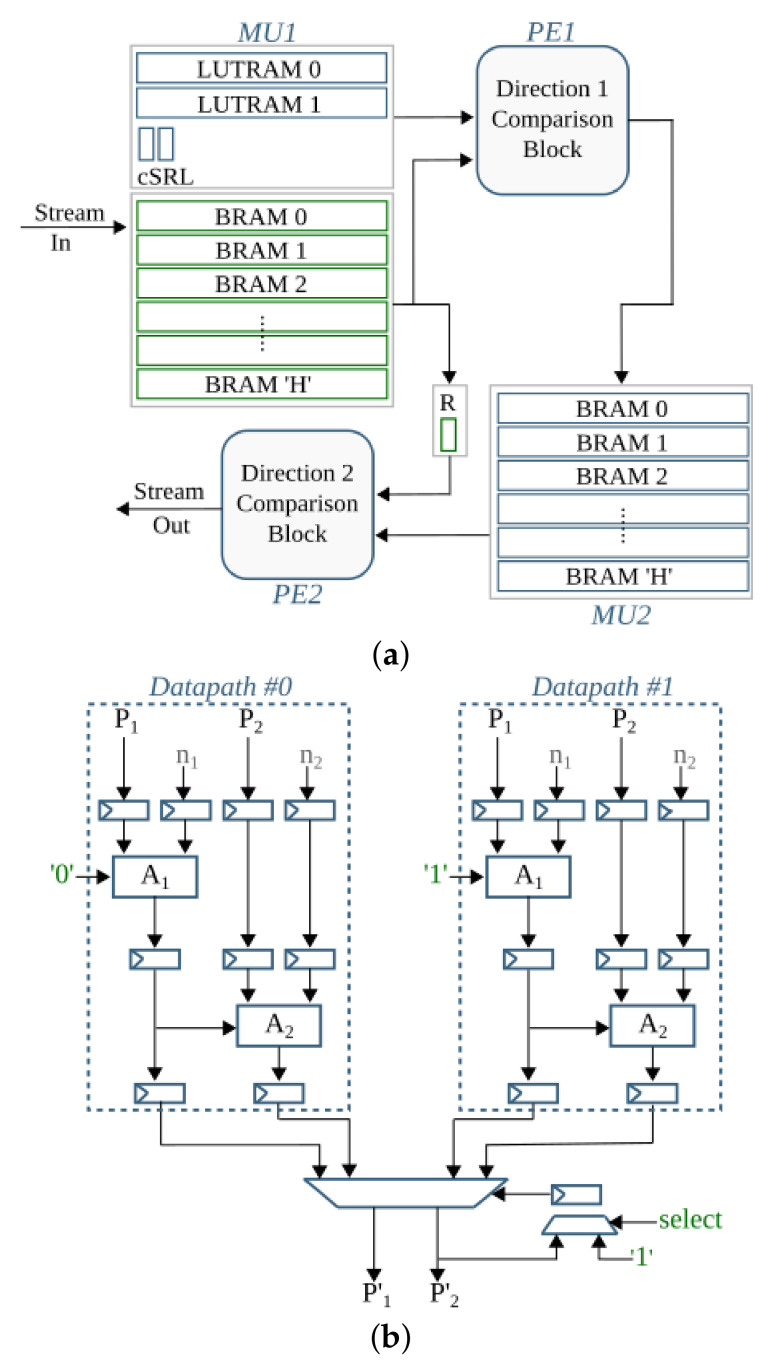
(**a**) hardware architecture of the alternate hole-fill algorithm. *MU1* and *MU2* are the memory units and *PE1* and *PE2* are the processing engines. (**b**) Datapath optimization. Here, $\left(P_{1},P_{2}\right)$ are input pixels read together, $\left(n_{1},n_{2}\right)$ are the corresponding neighbors. $\left(P_{1}^{{}^{\prime }},P_{2}^{{}^{\prime }}\right)$ are the corresponding output pixels. *Select* is used to input ′1′ for every set of pixels that start exactly at the boundary of the image.

The *hole-fill* operation is a binary pixel computation that adds pixels to the boundaries of objects to fill holes within the image. The largely iterative morphological reconstruction by erosion operation used in the original anyOCR is not feasible to implement in hardware. Hence, as described in Section 5.2.3, the connectivity-based *alternate hole-fill* operation is used for the *i*DocChip system that achieves sufficient hole-filling quality with only two image scans. As explained in [27], the *alternate hole-fill* algorithm first creates a binary mask image *M* using the input image dimensions and setting all but the border the pixel values to ${}^{\prime }1^{\prime }$. In hardware, this mask image is created on the fly. Then the pixels of the input image *I* and the neighborhood pixel values of the mask image for the sliding window $\left(k_{v}\times k_{h}\right)$ are analyzed as shown in Equation (8). In the *i*DocChip system, a cross-connective window Figure 17b, is used. After completely updating the mask image for the first run in the raster scan direction, the algorithm processes the anti-raster scan sequence similarly. The hardware architecture of this algorithm for the 2-direction sequence is shown in Figure 19a.

(8) $$M\left(i,j\right)=\left\{\begin{array}{cc} 0, & I\left(i,j\right)==0\;\;\;or\;\sum_{(k_{v},k_{h})\in K}M\left(k_{v},k_{h}\right)==0\\ M(i,j), & otherwise \end{array}\right.$$

The *i*DocChip system processes multiple pixels in each clock cycle. However, the *alternate hole-fill* algorithm is sequential in nature, where the result from the previous pixel affects the result of the following pixels. To overcome this issue and maximize throughput, multiple datapaths are used to process several pixels in parallel. A pipelined design based on a carry-select adder is shown in Figure 19b. The two parallel data paths compute a result under different assumption of the previous pixel result (i.e., 0 or 1). This architecture breaks the dependency structure of the *alternate hole-fill* design and supports high-throughput computations for streams with more than one incoming pixel. Moreover, the datapath has a parameterizable width; hence it can be scaled to process any number of pixels.

*Single-pass CCA* extracts features of interest for each distinct objects of an image while performing the connected component labeling task. In the *i*DocChip system, five *single-pass CCA* operations are implemented in hardware. These operations use a square-connective window; see Figure 17a. Due to the streaming architecture, only four neighboring pixels $(n_{1}$ to $n_{4})$ are processed per clock cycle. Similar to [80], the *single-pass CCA* gives the current pixel the smallest label found within the sliding window. Equivalent labels of a connected component are tracked using *index labels*, while its *root label*, i.e., the smallest representative of a component, is tracked using *root flag*. Root labels point to themselves by containing their own number as their index label. Moreover, a *data table* contains the desired features and characteristics of connected components. The three regular *single-pass CCAs* used in the text line extraction step of the *i*DocChip system compute commonly extracted features, such as *area*, *bounding box*, *width*, and *height*. However, the two *single-pass CCA* operations of the text and image segmentation pipeline require two images to process output. The initial operation finds *unique labels* after intersecting the two incoming images. Then, the subsequent *single-pass CCA* reuses both incoming images to recompute CCA and generate result pixels by indexing the *unique labels* and applying a *union* operation. The *single-pass CCA* operations that require recomputation use BRAMs to buffer input images and/or delay the faster stream line from the two incoming images as shown in Figure 12 and Figure 14. The *root flag*, *index label*, and *data table* are tracked, stored, and updated using dedicated line buffers.

#### 6.1.3. Computations with Irregular Memory Access

Algorithms that exhibit irregular memory access patterns show poor performance on hardware architectures, particularly when memory access latency is variable. These operations reduce the hardware throughput due to an unbalanced workload. The output stream irregularity then propagates through the subsequent operations. Different techniques have been used to mask the long latency caused by irregular memory access patterns, as described below.

**Figure 20:**
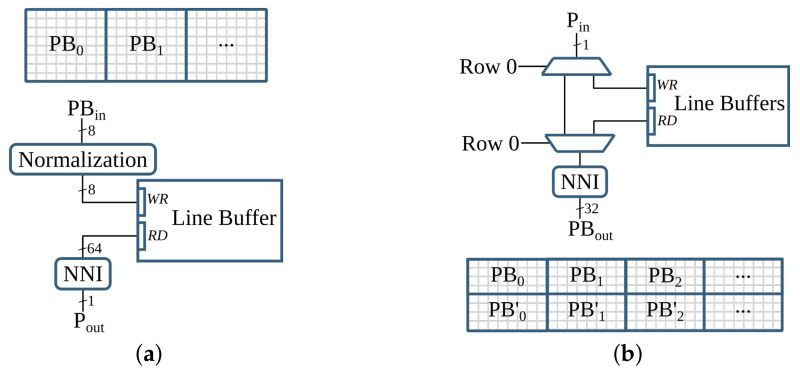
Custom memory access pattern for transferring pixel blocks (PBs) from external memory to the FPGA and hardware designs of (**a**) normalization and image downscale operations (**b**) image upscale operation using nearest-neighbor interpolation (NNI).

In the binarization step of *i*DocChip, two *nearest-neighbor interpolations* are implemented in hardware. To scale down the incoming image by a zoom factor of *z*, a block of z×z pixels is reduced to a single pixel. The hardware implementation of the nearest-neighbor interpolation requires to buffer *z* rows to produce a single output. In order to save valuable resources while avoiding the large latency, we use a custom memory access pattern, where the original input image of the binarization step is streamed from off-chip memory in a block of pixels instead of row by row, as shown in Figure 20a. Since the *normalization* core is *pixel-based* computation, out of order memory access pattern does not affect its result. Instead, the proposed memory access pattern regulates the interval at which the *percentile* filters receive pixels. After processing the *percentile* filters, the resulting image is scaled up using *nearest-neighbor interpolation*. This operation outputs multiple pixels in a block, refer to Figure 20b.

**Figure 21:**
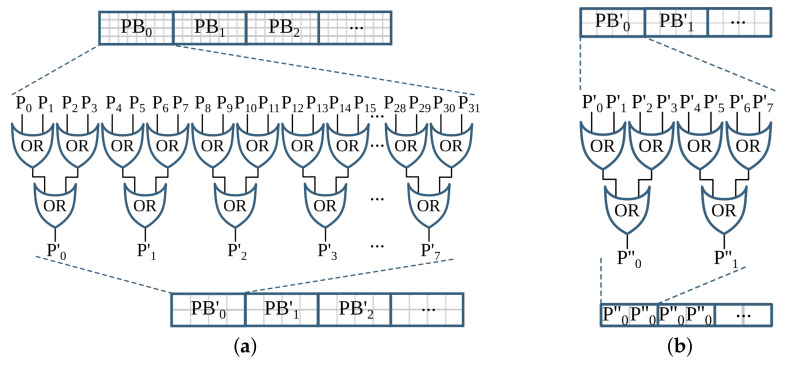
Hardware architecture for the two consecutive *reduction* operations. (**a**) first reduction with T=1 (**b**) second reduction with T=1.

After the second *nearest-neighbor interpolation*, the following operations of the binarization step are *pixel-based computations* that are not affected by the block processing (see Figure 10). Similarly, the *inversion* operation of the text and image segmentation pipeline inverts each pixel from the output pixel blocks of the binarization step. Then the two *reduction* operations of T=1 are applied to resize the incoming image by a factor of 4, as shown in Figure 21. At the end of this step, the pixel block is flattened into consecutive pixels, which are fed into the *alternate hole-fill* operation.

**Figure 22:**
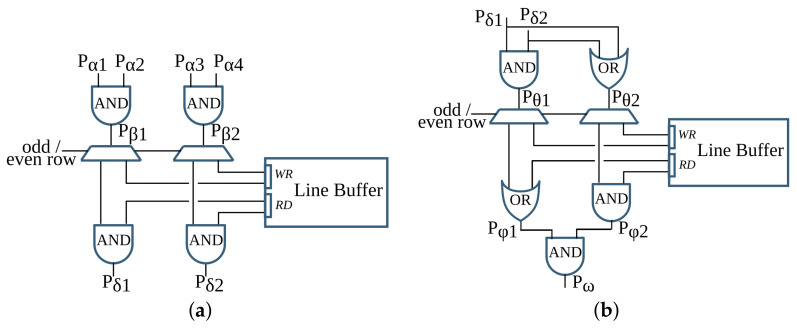
Architecture of the *reduction* operations with (**a**) with *T* = 4 and (**b**) with *T* = 3.

The *alternate hole-fill* operation uses multiple datapaths to generate multiple pixel outputs. Then the following two *reduction* operations of T=4 and T=3 are applied. These blocks require pixels of two rows to produce an output. Hence, *line buffers* are used to store row pixels, as shown in Figure 22. The irregularity of the output interval of the *reduction* operations propagates to the *opening* hardware block. However, this irregularity does not propagate further, as the *expansion* operations together with the image buffer (see Figure 12) resume the raster scan order of pixels.

The *reduction* operations of the text line extraction step with T=1 receive input images from the off-chip memory. Similar to the original grayscale input image, the memory access pattern is customized to stream block of pixels in order to avoid irregular output streams.

### 6.2. Hardware Architectures of Text Line Recognition

**Figure 23:**
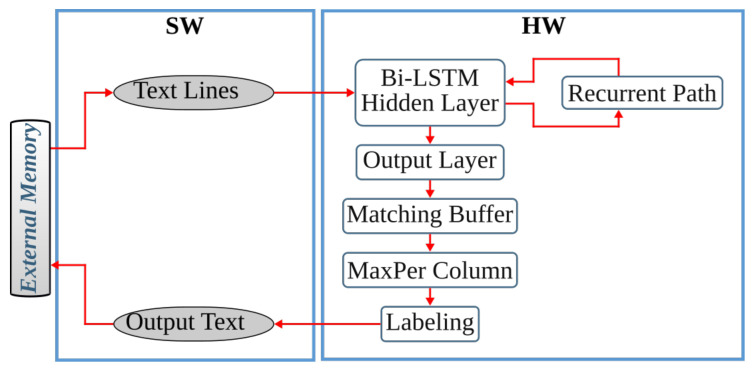
The system-level architecture of the *i*DocChip text line recognition step.

The main challenge to design an efficient architecture of an algorithm with a feedback loop comes from recurrency that becomes a throughput bottleneck. The next step, the column of a text line image, can be processed if the previous step has been finished due to a precedence constraint that stalls the pipeline part of the time. An efficient solution is to make the pipeline busy with calculations that do not have recurrent dependencies between each other. In the case of Bi-LSTM, the processing of the inputs from different directions is independent. We rearrange memory access patterns and propose an architecture that processes the image with the forward and the backward columns interleaved. First, the architecture processes the first column from the forward direction, then the first column from the backward direction, then the second column, etc. This approach keeps the pipeline always busy without throughput penalties if the number of sequentially processed LSTM cells is sufficient. In the following, we describe a hardware architecture of the text line recognition step depicted in Figure 23.

**Figure 24:**
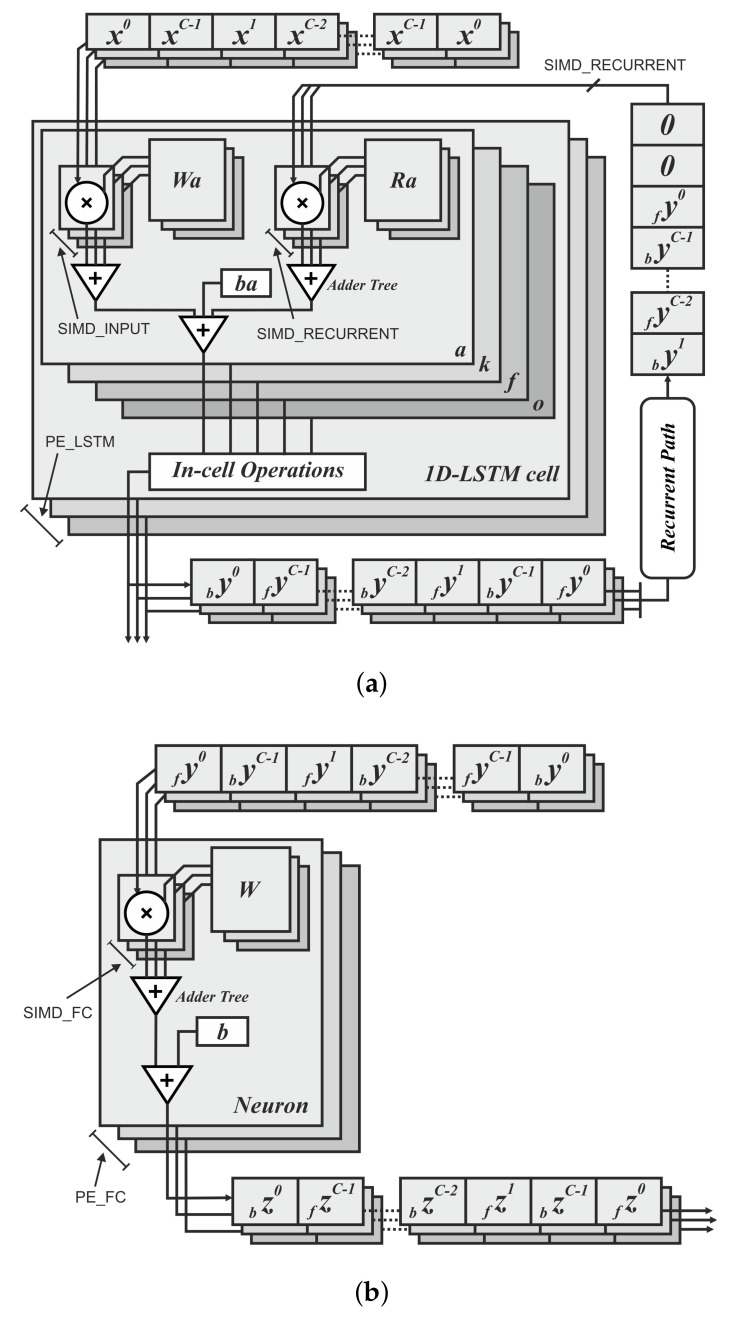
(**a**) Hardware design of the BiLSTM layer. (**b**) Hardware design of the Output Layer.

The *Bi-LSTM Hidden Layer* module implements the Bi-LSTM layer with parametrizable parallelization as depicted in Figure 24a, where $x^{t}$ is an input activation corresponding to column *t* with (t∈0⋯C−1), ${}_{f}y^{t}$ and ${}_{b}y^{t}$ are output/recurrent activations corresponding to the forward and backward processing directions, respectively. W and R are weight matrices, while b is a bias corresponding to the gates of LSTM cell (a,k,f,o). The parametrizable architecture allows for the application of coarse-grained parallelization on a level of LSTM cells and fine-grained parallelization on a level of dot products with all LSTM’s gates instantiated. The former, indicated as PE_LSTM unrolling allows the concurrent execution of different LSTM cells, while the latter, indicated as SIMD_INPUT and SIMD_RECURRENT, folds the execution of a single cell in multiple cycles. PE and SIMD stand for Processing Element and Single Instruction, Multiple Data, respectively. This flexibility allows for tailoring parallelism according to the required latency and throughput. Using the proposed memory access pattern, only a half of memory bandwidth and computing resources are required compared to the duplicated datapath due to bidirectional LSTM. However, a doubling of weights’ memory is unavoidable. The *Recurrent Path* module converts the output from the hidden layer that has a width that is a multiple of PE_LSTM into input with a width that is a multiple of SIMD_RECURRENT.

The *Output Layer* module implements the dense layer folded with batch normalization; see Figure 24b, where ${}_{f}z^{t}$ and ${}_{b}z^{t}$ are output activations corresponding to the forward and backward processing directions. W is a weight matrix, and b is a bias corresponding to a neuron. Conventionally, the Output Layer processes the concatenated output sequences from the forward hidden layer and the backward hidden layer taken in reverse order. In this case, it requires waiting for $2\times N^{H}\times C$ clock cycles before all outputs from the hidden layers are available and $2\times N^{H}\times C$ memory entries to store the outputs. We propose to start processing as soon as the outputs from the forward hidden layer are available. As a result, we avoid implementing a large buffer. The *Output Layer* is implemented with a coarse-grained parallelization on a level of neurons denoted as PE_FC, and fine-grained parallelization on a level of dot products denoted as SIMD_FC equal to PE_LSTM of the previous hidden layer to match the throughput.

**Figure 25:**
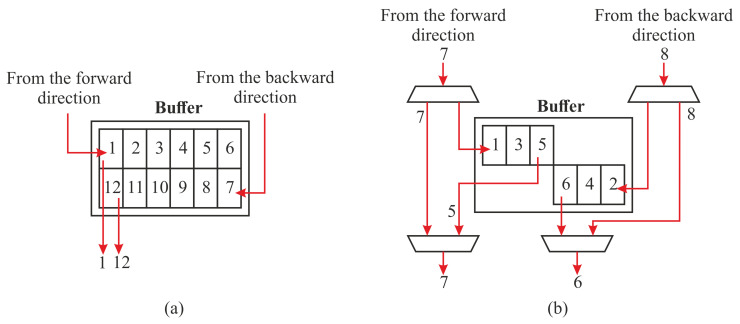
Writing and reading patterns: (**a**) the conventional approach on general-purpose platforms, (**b**) the proposed approach that halves the memory and time. The numbers indicate the writing order assuming, e.g., 6 columns per image.

The *Matching Buffer* is a hardware module used to store and align the outputs from the *Output Layer*. The outputs from the *Output Layer* corresponding to the forward and the backward hidden layer from the same columns have to be summed up and that requires buffering of the half-sums. Without the proposed memory access pattern, the algorithm would require storing of $2\times H^{O}\times C$ values; see Figure 25a. In contrast, in the proposed architecture, the required memory is reduced to half. As soon as the last value corresponding to the centric column from the backward direction has been written to the memory, we stop writing to the memory and start reading the values from the *Output Layer* corresponding to the forward direction from the centric column; see Figure 25b. This way, we reduce the size of the required buffer and processing time to half. In the end, the half-sums are summed and forwarded to the next module.

The *MaxPerColumn* module finds a label with the highest value per column and forwards its index and corresponding value to the *Labeling* module, which is implemented according to the algorithm explained in Section 3.4.

## 7. Experimental Setup and Results

To evaluate the heterogeneous hardware-software architecture of the optimized *i*DocChip system, it is implemented and compared with different software-based implementations. As stated previously, in the experiments, we use the historical Latin document images dataset [25]. For our experiments, we use two versions of the dataset: (1) high-resolution scanned images of 400ppi and (2) lower resolution images of 72ppi taken by smartphone camera (Samsung Galaxy A9).

To provide a comparison to modern commercially available OCR solutions, we compare character-level accuracy achieved by the *i*DocChip and Cloud Vision OCR from Google, using high- and low-resolution images. As shown in Table 3, the accuracy achieved by *i*DocChip is higher in both cases. The better accuracy of *i*DocChip is explained by the particular focus of the system to transcribe historical documents. Most of the character errors of the Cloud Vision OCR occur due to falsely detected text in the image area, which is related to specificity of the illustrations in the particular document images.

### 7.1. The *i*DocChip Hardware Accelerator

For the *i*DocChip hardware accelerator, the IP blocks of the operations offloaded to hardware are designed using Xilinx^®^ Vivado^®^ High-Level Synthesis version 2018.1. The complete system is implemented using Vivado block design targeting Zynq^®^-7000 All Programmable SoC, specifically Zynq 7045, which features xc7z045ffg900-2 FPGA fabric and dual-core ARM^®^ Cortex™-A9 processor. The implementation of the end-to-end *i*DocChip system is evaluated and tested on Zynq^®^-7000 SoC ZC706 board, which contains the target device. The acquired dataset images are transferred to the dynamic random-access memory (DRAM) of the ZC706 board.

**Figure 26:**
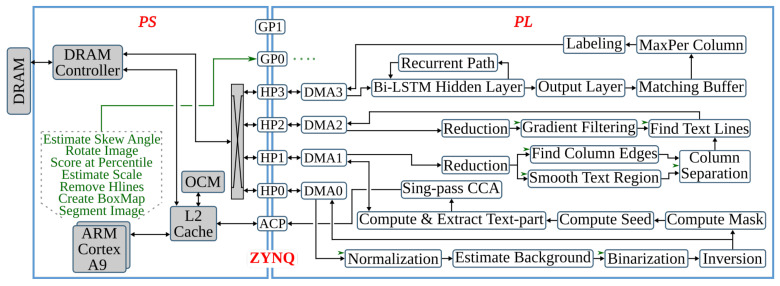
Zynq implementation of *Text and Image Segmentation* hardware architecture.

The ARM CPU runs Linaro Ubuntu Linux version 16.1. The software-programmable parameters, such as kernel values of filters, are transferred from the PS through the general-purpose input/output (GPIO) ports using an AXI-Lite interface. Custom DMAs are implemented, which are responsible for the reads/writes of data from/to DRAM using AXI memory-mapped non-coherent interface and converting each transaction into AXI stream. The DMAs transfer data in bursts of 64 bits. The DMAs enable overlapping of computations with memory transfers. The software parts of the heterogeneous *i*DocChip architecture that run on the ARM Cortex-A9 processor cores of the Zynq 7045 device are implemented in C/C++. These CPU cores run at 800 MHz, while the FPGA fabric runs at 166 MHz. The system block diagram of *i*DocChip implemented on Zynq is shown in Figure 26.

The total resource consumption of the hardware accelerator of *i*DocChip implemented on Xilinx Zynq 7045 is given in Table 4. Compared to the total utilization of the previous separately implemented pipeline steps [26,27,28,29], the end-to-end accelerator utilizes on average twice more resources. The higher resource consumption is because in the end-to-end implementation (1) we have used higher parallelism for some blocks, (2) the hardware-software partitioning has been changed that resulted in more functions to be implemented in hardware, (3) some blocks support higher parameterization, and (4) coupling the four separate pipeline steps together demands extra routing resources.

### 7.2. Comparisons between the Hardware and Software Implementations

The original anyOCR is a Python-based software that uses the multi-dimensional image processing library [84] and runs on a multi-threaded Intel^®^ Core™ i7-4790T with Turbo Boost up to 3.9 GHz for one active core and 2.7 GHz for four active cores. For further analysis, the runtime and energy efficiency of the optimized software implementations are examined on different platforms; see Table 5.

#### 7.2.1. Software Optimizations

For a fair comparison of the software implementations against the hardware design, different platform-dependent and algorithmic optimizations are performed to accelerate the OCR pipeline. Additionally, the Python implementation of the reference anyOCR chain is optimized in a similar manner to the *i*DocChip algorithm. For the multi-threaded C/C++ implementations, an image-level coarse-grained parallelization is applied using OpenMP API. As a result, all available threads of the given CPU process separate images at each point in time. This coarse-grained approach has appeared to be more efficient than fine-grained parallelization applied on a level of functions or loops because it does not require any inter-core or inter-thread synchronization. Dynamic scheduling in OpenMP is used to avoid idling threads, such that the threads start processing the next image right when they finish the current image. Moreover, hyper-threading is used in the case of Intel CPU. All software implementations are compiled with GCC 7.4.0 and -O3 optimization flag.

#### 7.2.2. Energy Consumption

The processors used for comparison have hardware setups with peripherals and extra components that contribute to the overall power consumption. For a fair comparison of power and energy consumption among the different platforms, we consider only dynamic power consumption ($P_{dyn}$) that exhibits minimal influence from components that do not contribute to the computation. To compute $P_{dyn}$, we use equation Equation (9). The $P_{dyn}$ is calculated by subtracting the idle power $P_{idle}$ from the power consumption of the complete system during the processing of all the images in the dataset, $P_{complete}$. The consumed energy $E_{cons}$ is computed from the average dynamic power $P_{dyn}$, as shown in Equation (10).

(9) $$P_{dyn}=\left(P_{complete}-P_{idle}\right)$$

(10) $$E_{cons}=P_{dyn}\times Runtime$$

The idle power consumption of the processor systems is measured without any workload. By subtracting this value, the influence of the unwanted power consumption of the extra hardware peripherals is minimized. The $E_{cons}$, however, includes the unavoidable energy consumption for the extra cooling caused by intense computations. Similarly for Zynq, $P_{idle}$ is the power consumption of the complete board while CPU is idle and FPGA is not configured. The power has been measured physically using digital wall socket power meter Voltcraft^®^ VC-870.

### 7.3. Results and Discussion

**Figure 27:**
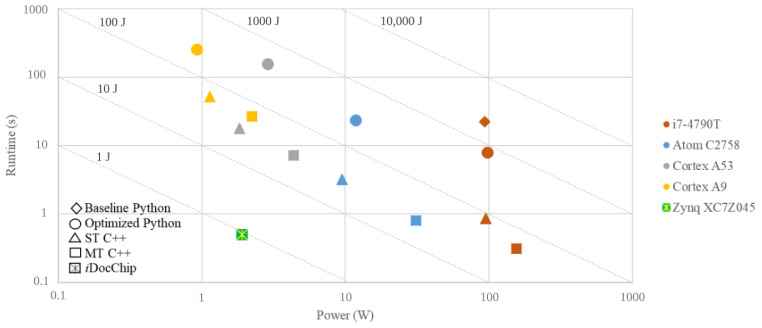
Power vs. runtime comparisons of the reference anyOCR and the optimized *i*DocChip algorithm on different platforms. Runtime is given per image. Single-threaded and multi-threaded implementations are represented as *ST* and *MT*. The grid lines show energy consumption in Joules (J).

**Figure 28:**
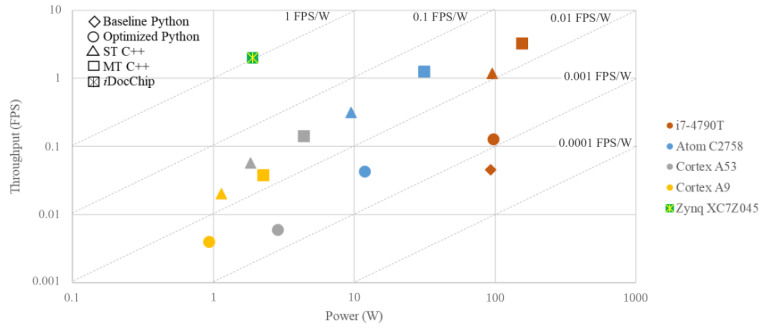
Power vs. FPS comparisons of the reference anyOCR and the optimized *i*DocChip algorithm on different platforms. The grid lines show energy efficiency in FPS/W.

Figure 27 shows comparisons of the *i*DocChip design and software implementations of the anyOCR running on different platforms in terms of runtime and power. The hybrid hardware-software implementation on FPGA provides a speedup of more than 44× and 15× compared to the baseline and optimized anyOCR implementations running on i7-4790T, respectively. The multi-threaded C/C++ implementation of the algorithm running on the same processor has the highest performance that is 1.6× higher than the hardware implementation. However, it also exhibits the highest power, which is **82×** more than the FPGA. The embedded CPUs, Cortex A53, and Cortex A9 lag in performance even when running the highly optimized software implementation, resulting in 14× and 53× slower run-times compared to the iDocChip, respectively. Moreover, their energy consumption is higher than the hardware solution, namely 63× for Cortex A53 and 32× for Cortex A9. Moreover, the FPGA implementation has the highest energy efficiency providing >1 FPS/W, as shown in Figure 28. The Intel Core i7-4790T CPU running the single- or multi-threaded C/C++ implementation and the multi-threaded Atom implementation provide sufficient throughput (above 1FPS), however, at the expense of high power consumption. In contrast, the single-threaded embedded CPUs achieve the power requirements consuming less than 2 W; however, they fail to meet the throughput requirements.

Revisiting the system design specifications detailed in Section 1, the heterogeneous end-to-end *i*DocChip system achieves the goal for an energy-efficient portable device with 1 FPS/W under the constrained power budget of 2 W. Furthermore, with a throughput of 2 FPS, the design has a real-time processing latency of 500 ms per image. Hence, the *i*DocChip system meets all design constraints.

The presented accelerator provides a unique platform for testing various document image processing algorithms in hardware, due to its reconfigurability and flexibility. The parameters of the algorithms can be adjusted for new datasets or even the complete steps can be replaced with newer algorithms without disturbing the integrity of the complete solution.

## 8. Conclusions

In this paper, we presented a heterogeneous hardware-software architecture for an end-to-end optical character recognition system along with various highly optimized software implementations. Based on the new architecture, we implemented a heterogeneous accelerator on Zynq 7045. The resulting hybrid system outperforms the original software implementation running on i7-4790T by a factor of 44 in terms of runtime and by a factor of 2201 with respect to energy efficiency. For further analysis, our design is compared with other platforms running the optimized software implementations with respect to runtime, power, and energy efficiency. Our device achieves a throughput of 2 FPS for an image with 2166×3219 size while exhibiting a power consumption of 1.9 W and energy efficiency of 1 J per image. None of the considered CPUs fulfill the power (≤2 W) and real-time (≥2 FPS) requirements at the same time. In contrast, our accelerator meets all design requirements for an energy-efficient portable OCR device. Hence, we conclude that the presented computer vision and image processing algorithms benefit from being migrated to our dedicated accelerator.

## Figures and Tables

**Table 1:** Character-level accuracy for binarization using U-Net based and PBB methods.

Binarization Method	Model [Mparams]	“Narrenschiff” Character-Level Accuracy [%]
State-of-the-art U-Net [74]	93.1	75.05
Low-complexity U-Net [73]	0.76	74.73
Hand-tuned percentile-based	-	76.30

**Table 2:** Algorithmic optimization effects on system accuracy (for test dataset [25]).

Original anyOCR Accuracy		76.3%
**Modification Type**	**Modified Operations**	**Accuracy**
BIN-1	After relocating *skew angle computation*	76.42%
BIN-2	After relocating *image rotation* operation	76.54%
BIN-3	After relocating *high- and low-score calculations*	76.51%
BIN-4	After replacing *spline interpolations* by *nearest-neighbor*	76.65%
BIN-5	After increasing *zoom* value	76.34%
BIN-6	After modifying *percentile filter* operations	76.22%
TISEG-1	After using *alternate hole-fill* operation instead of *morphological reconstruction*	76.24%
TLEXT-1	After changing *find maximum pixel* computations by constant thresholds	75.23%
TLEXT-2	After changing *topological sort* algorithm to a *quick sort* operation	79.13%
TLEXT-3	After modifying the *propagate label* algorithm	79.24%
TLEXT-4	After adding a 4x *reduction* operation with T=1 and a 4x *expansion* operation	80.1%

**Table 3:** Character-level accuracy of Cloud Vision OCR and *i*DocChip OCR.

	High-Resolution Images, Accuracy [%]	Low-Resolution Images, Accuracy [%]
Cloud Vision OCR, Google	76.32	76.39
*i*DocChip OCR	80.10	79.82

**Table 4:** Resource utilization of the hardware implementation for the end-to-end OCR *i*DocChip system (this work) compared to the total resource utilization of the previous separately implemented pipeline steps [26,27,28,29] using Zynq 7045 device @ 166MHz.

Pipeline	LUT	FF	BRAM 36 Kb	DSP
Total of previous works	109,701 (51%)	101,179 (24%)	248 (46%)	99 (11%)
End-to-end OCR	201,895 (93%)	323,067 (74%)	512 (94%)	129 (15%)
Available	218,600	437,200	545	900

**Table 5:** Configurations of different platforms for software based tests.

Platform	Num. Cores	Threads per Core	Total Threads	Freq. [GHz]	Tested on			
					Python Baseline	Python Optimized	C++ (ST)	C++ MT
i7 4790T	4	2	8	2.7	✓	✓	✓	✓
Atom C2758	8	1	8	2.4		✓	✓	✓
Cortex A53	4	1	4	1.5		✓	✓	✓
Cortex A9	2	1	2	0.8		✓	✓	✓

## Abbreviations

ASIC	application-specific integrated circuit
AXI	advanced extensible interface
Bi-LSTM	Bidirectional LSTM
BRAM	block random-access memory
CC	connected component
CCA	connected component analysis
CCL	connected component labeling
CER	character error rate
CMOS	complementary metal oxide semiconductor
CNN	Convolutional Neural Network
CPU	central processing unit
CTC	connectionist temporal classification
DIBCO	Document Image Binarization Competition
DMA	direct memory access
DRAM	dynamic random-access memory
EDT	Euclidean Distance Transform
ELM	Extreme Learning Machine
FPGA	field-programmable gate array
FPS	frames per second
GPIO	general-purpose input/output
GPU	graphics processing unit
IP	intellectual property
LSTM	long short-term memory
MD-LSTM	multidimensional long short-term memory
OCR	optical character recognition
PBB	percentile-based binarization
PL	programmable logic
PS	processing system
SE	structuring element
SIPO	Serial In - Parallel Out
SoC	System-on-Chip
SVM	support-vector machine
TDP	thermal design power

## References

[1] PenPower http://www.penpowerinc.com.

[2] Scanning Pens https://www.scanningpens.com/.

[3] Scanmaker https://scanmarker.com/.

[4] Ectaco C-Pen. https://www.ectaco.com/cpen-30/.

[5] IRISPen. https://www.irislink.com/EN-US/c1870/Compare-IRIS-digital-pens.aspx.

[6] C-PEN. https://cpen.com/.

[7] Google Cloud Vision OCR. https://cloud.google.com/vision/docs/ocr.

[8] Microsoft Computer Vision. https://azure.microsoft.com/en-us/services/cognitive-services/computer-vision/.

[9] ABBYY Cloud OCR. https://www.abbyy.com/cloud-ocr-sdk/.

[10] CloudOCR. https://cloudocr.com/.

[11] Forbes-FPGA Chip on iPhone 7. https://www.forbes.com/sites/aarontilley/2016/10/17/iphone-7-fpga-chip-artificial-intelligence/?sh=6fbb634d3c69.

[12] Vuzix Glass OCR. https://www.vuzix.com/appstore/app/glass-ocr-for-m300.

[13] ORCAM OCR Device to Wear on Glasses. https://www.orcam.com/en/media/life-changing-optical-character-recognition-glasses/.

[14] Envision Glasses. https://www.letsenvision.com/envision-glasses.

[15] eSight. https://esighteyewear.com/.

[16] ABBYY https://www.abbyy.com/en-eu/.

[17] Omnipage. https://www.kofax.com/Products/omnipage?source=nuance.

[18] OCRopus. https://github.com/ocropus/ocropy.

[19] Tesseract. https://github.com/tesseract-ocr.

[20] Bukhari S.S., Kadi A., Jouneh M.A., Mir F.M., Dengel A. (2017). anyOCR: An Open-Source OCR System for Historical Archives. Proceedings of the 2017 14th IAPR International Conference on Document Analysis and Recognition (ICDAR).

[21] Narragonien-Digital. http://www.narragonien-digital.de/exist/home.html.

[22] Kallimachos. http://kallimachos.de/kallimachos/index.php/Projektbeschreibung.

[23] German Research Centre for Artificial Intelligence (DFKI) https://www.dfki.de/web/news/detail/News/any-ocr/.

[24] University of Würzburg https://www.uni-wuerzburg.de/aktuelles/einblick/single/news/narrenschi/.

[25] Narrenschif. http://kallimachos.de/kallimachos/index.php/Narragonien.

[26] Rybalkin V., Bukhari S.S., Ghaffar M.M., Ghafoor A., Wehn N., Dengel A. (2018). iDocChip: A Configurable Hardware Architecture for Historical Document Image Processing: Percentile Based Binarization. Proceedings of the ACM Symposium on Document Engineering 2018.

[27] Tekleyohannes M.K., Rybalkin V., Ghaffar M.M., Varela J.A., Wehn N., Dengel A. (2021). iDocChip: A Configurable Hardware Architecture for Historical Document Image Processing. Int. J. Parallel Program..

[28] Tekleyohannes M.K., Rybalkin V., Ghaffar M.M., Wehn N., Dengel A. (2019). iDocChip-A Configurable Hardware Architecture for Historical Document Image Processing: Text Line Extraction. Proceedings of the 2019 International Conference on ReConFigurable Computing and FPGAs (ReConFig).

[29] Rybalkin V., Wehn N., Yousefi M.R., Stricker D. (2017). Hardware architecture of bidirectional long short-term memory neural network for optical character recognition. Proceedings of the Conference on Design, Automation & Test in Europe.

[30] Tekleyohannes M.K., Rybalkin V., Bukhari S.S., Ghaffar M.M., Varela J.A., Wehn N., Dengel A. iDocChip—A Configurable Hardware Architecture for Historical Document Image Processing: Multiresolution Morphology-based Text and Image Segmentation. Proceedings of the 6th International Embedded Systems Symposium (IESS).

[31] Brugger C., Dal’Aqua L., Varela J.A., De Schryver C., Sadri M., Wehn N., Klein M., Siegrist M. (2015). A quantitative cross-architecture study of morphological image processing on CPUs, GPUs, and FPGAs. Proceedings of the 2015 IEEE Symposium on Computer Applications & Industrial Electronics (ISCAIE).

[32] Qasaimeh M., Denolf K., Lo J., Vissers K., Zambreno J., Jones P.H. (2019). Comparing Energy Efficiency of CPU, GPU and FPGA Implementations for Vision Kernels. Proceedings of the 2019 IEEE International Conference on Embedded Software and Systems (ICESS).

[33] Page A., Mohsenin T. (2013). An efficient & reconfigurable FPGA and ASIC implementation of a spectral Doppler ultrasound imaging system. Proceedings of the 2013 IEEE 24th International Conference on Application-Specific Systems, Architectures and Processors.

[34] Jiang S., He D., Yang C., Xu C., Luo G., Chen Y., Liu Y., Jiang J. (2018). Accelerating mobile applications at the network edge with software-programmable fpgas. Proceedings of the IEEE INFOCOM 2018-IEEE Conference on Computer Communications.

[35] Bonamy R., Bilavarn S., Muller F., Duhem F., Heywood S., Millet P., Lemonnier F. (2018). Energy efficient mapping on manycore with dynamic and partial reconfiguration: Application to a smart camera. Int. J. Circuit Theory Appl..

[36] Xilinx, Inc Zynq®-7000 All Programmable SoC. https://www.xilinx.com/products/silicon-devices/soc/zynq-7000.html.

[37] Baidu’s Apollo Driverless Platform. https://www.electronicdesign.com/markets/automotive/article/21119589/xilinx-soc-fpga-powers-baidus-apollo-driverless-platform.

[38] Topic Embedded Systems. https://topic.nl/en/products.

[39] AXIOM Beta: A Professional Digital Cinema Camera. https://apertus.org/axiom.

[40] Ishikawa S.N., Takahashi T., Watanabe S., Narukage N., Miyazaki S., Orita T., Takeda S., Nomachi M., Fujishiro I., Hodoshima F. (2018). High-speed X-ray imaging spectroscopy system with Zynq SoC for solar observations. Nucl. Instrum. Methods Phys. Res. Sect. A Accel. Spectrom. Detect. Assoc. Equip..

[41] Mata-Carballeira Ó., Gutiérrez-Zaballa J., del Campo I., Martínez V. (2019). An FPGA-Based Neuro-Fuzzy Sensor for Personalized Driving Assistance. Sensors.

[42] Guo K., Sui L., Qiu J., Yu J., Wang J., Yao S., Han S., Wang Y., Yang H. (2017). Angel-Eye: A complete design flow for mapping CNN onto embedded FPGA. IEEE Trans. Comput.-Aided Des. Integr. Circuits Syst..

[43] Afroge S., Ahmed B., Mahmud F. (2016). Optical character recognition using back propagation neural network. Proceedings of the 2016 2nd International Conference on Electrical, Computer & Telecommunication Engineering (ICECTE).

[44] Wei T.C., Sheikh U., Ab Rahman A.A.H. (2018). Improved optical character recognition with deep neural network. Proceedings of the 2018 IEEE 14th International Colloquium on Signal Processing & Its Applications (CSPA).

[45] Nasien D., Haron H., Yuhaniz S.S. (2010). Support Vector Machine (SVM) for English handwritten character recognition. Proceedings of the 2010 Second International Conference on Computer Engineering and Applications.

[46] Lavanya K., Bajaj S., Tank P., Jain S. (2017). Handwritten digit recognition using hoeffding tree, decision tree and random forests—A comparative approach. Proceedings of the 2017 International Conference on Computational Intelligence in Data Science (ICCIDS).

[47] Ilmi N., Budi W.T.A., Nur R.K. (2016). Handwriting digit recognition using local binary pattern variance and K-Nearest Neighbor classification. Proceedings of the 2016 4th International Conference on Information and Communication Technology (ICoICT).

[48] Sampath A., Gomathi N. (2017). Decision tree and deep learning based probabilistic model for character recognition. J. Cent. South Univ..

[49] Younis K.S., Alkhateeb A.A. A new implementation of deep neural networks for optical character recognition and face recognition. Proceedings of the New Trends in Information Technology.

[50] Srivastava S., Priyadarshini J., Gopal S., Gupta S., Dayal H.S. (2019). Optical character recognition on bank cheques using 2D convolution neural network. Applications of Artificial Intelligence Techniques in Engineering.

[51] Das T., Tripathy A.K., Mishra A.K. (2017). Optical character recognition using artificial neural network. Proceedings of the 2017 International Conference on Computer Communication and Informatics (ICCCI).

[52] Moysset B., Kermorvant C., Wolf C., Louradour J. (2015). Paragraph text segmentation into lines with recurrent neural networks. Proceedings of the 2015 13th International Conference on Document Analysis and Recognition (ICDAR).

[53] Murdock M., Reid S., Hamilton B., Reese J. (2015). ICDAR 2015 competition on text line detection in historical documents. Proceedings of the 2015 13th International Conference on Document Analysis and Recognition (ICDAR).

[54] Kundu S., Paul S., Bera S.K., Abraham A., Sarkar R. (2020). Text-line extraction from handwritten document images using GAN. Expert Syst. Appl..

[55] Breuel T.M., Ul-Hasan A., Al-Azawi M.A., Shafait F. (2013). High-performance OCR for printed English and Fraktur using LSTM networks. Proceedings of the 2013 12th International Conference on Document Analysis and Recognition.

[56] Singh B.M., Sharma R., Mittal A., Ghosh D. (2011). Parallel implementation of Souvola’s binarization approach on GPU. Int. J. Comput. Appl..

[57] Chen X., Lin L., Gao Y. (2016). Parallel nonparametric binarization for degraded document images. Neurocomputing.

[58] Singh B.M., Sharma R., Mittal A., Ghosh D. (2011). Parallel implementation of Otsu’s binarization approach on GPU. Int. J. Comput. Appl..

[59] Soua M., Kachouri R., Akil M. (2018). GPU parallel implementation of the new hybrid binarization based on Kmeans method (HBK). J. Real-Time Image Process..

[60] Westphal F., Grahn H., Lavesson N. (2018). Efficient document image binarization using heterogeneous computing and parameter tuning. Int. J. Doc. Anal. Recognit. (IJDAR).

[61] Sultana A., Meenakshi M. (2011). Design and development of fpga based adaptive thresholder for image processing applications. Proceedings of the 2011 IEEE Recent Advances in Intelligent Computational Systems.

[62] Rybalkin V., Wehn N. When Massive GPU Parallelism Ain’t Enough: A Novel Hardware Architecture of 2D-LSTM Neural Network. Proceedings of the 2020 ACM/SIGDA International Symposium on Field-Programmable Gate Arrays.

[63] Kumar A., Rastogi P., Srivastava P. (2015). Design and FPGA Implementation of DWT, Image Text Extraction Technique. Procedia Comput. Sci..

[64] Bai X., Shi B., Zhang C., Cai X., Qi L. (2017). Text/non-text image classification in the wild with convolutional neural networks. Pattern Recognit..

[65] Vignesh O., Mangalam H., Gayathri S. (2019). FPGA architecture for text extraction from images. Clust. Comput..

[66] Sanni K., Garreau G., Molin J.L., Andreou A.G. FPGA implementation of a Deep Belief Network architecture for character recognition using stochastic computation. Proceedings of the 2015 49th Annual Conference on Information Sciences and Systems (CISS).

[67] LeCun Y., Bottou L., Bengio Y., Haffner P. (1998). Gradient-based learning applied to document recognition. Proc. IEEE.

[68] Zho H., Zhu G., Peng Y. (2016). A RMB optical character recognition system using FPGA. Proceedings of the 2016 IEEE International Conference on Signal and Image Processing (ICSIP).

[69] De Oliveira Junior L.A., Barros E. (2018). An fpga-based hardware accelerator for scene text character recognition. Proceedings of the 2018 IFIP/IEEE International Conference on Very Large Scale Integration (VLSI-SoC).

[70] Ronneberger O., Fischer P., Brox T. (2015). U-net: Convolutional networks for biomedical image segmentation. International Conference On Medical Image Computing and Computer-Assisted Intervention.

[71] Pratikakis I., Zagoris K., Barlas G., Gatos B. (2017). ICDAR2017 competition on document image binarization (DIBCO 2017). Proceedings of the 2017 14th IAPR International Conference on Document Analysis and Recognition (ICDAR).

[72] Bezmaternykh P.V., Ilin D.A., Nikolaev D.P. (2019). U-Net-bin: Hacking the document image binarization contest. Comput. Opt..

[73] Karpinski R., Belaïd A. (2018). Combination of Two Fully Convolutional Neural Networks for Robust Binarization. Asian Conference on Computer Vision.

[74] Huang X., Li L., Liu R., Xu C., Ye M. (2020). Binarization of degraded document images with global-local U-Nets. Optik.

[75] Hu J., Shen L., Sun G. Squeeze-and-excitation networks. Proceedings of the IEEE Conference on Computer Vision and Pattern Recognition.

[76] Wagner R.A., Fischer M.J. (1974). The string-to-string correction problem. J. ACM (JACM).

[77] Bailey D.G., Johnston C.T. Single pass connected components analysis. Proceedings of the Image and Vision Computing.

[78] Bailey D.G. (2011). Design for Embedded Image Processing on FPGAs.

[79] Ma N., Bailey D.G., Johnston C.T. (2008). Optimised single pass connected components analysis. Proceedings of the 2008 International Conference on Field-Programmable Technology.

[80] Klaiber M.J. (2016). A Parallel and Resource-Efficient Single Lookup Connected Components Analysis Architecture for Reconfigurable Hardware. Ph.D. Thesis.

[81] Spagnolo F., Perri S., Corsonello P. (2019). An efficient hardware-oriented single-pass approach for connected component analysis. Sensors.

[82] Tekleyohannes M., Sadri M., Weis C., Wehn N., Klein M., Siegrist M. (2017). An advanced embedded architecture for connected component analysis in industrial applications. Proceedings of the Design, Automation & Test in Europe Conference & Exhibition (DATE).

[83] Tekleyohannes M.K., Weis C., Wehn N., Klein M., Siegrist M. (2018). A Reconfigurable Accelerator for Morphological Operations. Proceedings of the 2018 IEEE International Parallel and Distributed Processing Symposium Workshops (IPDPSW).

[84] Multi-Dimensional Image Processing (Scipy.Ndimage). https://docs.scipy.org/doc/scipy-0.14.0/reference/ndimage.html.

